# Slimehead Size Through Time: Testing the Temperature–Size Relationship in Late Cretaceous Trachichthyidae

**DOI:** 10.1002/ece3.72026

**Published:** 2025-09-25

**Authors:** Chloe V. Griffiths, James D. Witts, Julie C. S. Brown, Emma L. Bernard, Richard J. Twitchett

**Affiliations:** ^1^ Natural History Museum London UK; ^2^ University College London London UK

**Keywords:** body size, climate change, fish, fossil, temperature

## Abstract

As global temperatures rise, fish are predicted to become smaller. Body size is a fundamental trait that impacts many aspects of an animal's life history and ecology, and understanding how it may respond to climate change in particular fish groups, especially commercial or keystone species, is critical. The slimeheads (Family Trachichthyidae) include several commercially important species, but because they are deep‐dwelling, long‐lived fish that reproduce slowly, directly testing the temperature–size relationship in this family is challenging. Fortunately, Trachichthyidae have a long evolutionary history beginning in the Cretaceous, and their fossil record provides empirical data on the response of this family to past climate change events. In this study, we leveraged the extensive fossil record of the Late Cretaceous trachichthyid genus *Hoplopteryx* from the British Chalk Group of southern England, United Kingdom, to test whether its size declined at higher temperatures. Standard Lengths were measured from complete individuals and estimated from partial remains. Seawater palaeotemperature estimates were derived from oxygen stable isotope values (𝛿^18^O) of the bulk chalk rock surrounding the fossils using standard techniques and assumptions. Individual fish ranged from 56.3 to 262.6 mm in length, and measured seawater temperature estimates ranged from 19.5°C to 27.1°C. Multiple linear regression analyses revealed that the estimated seawater temperature was a significant negative predictor of Standard Length in the most common species, *Hoplopteryx lewesiensis*, supporting the prediction that higher temperatures led to smaller body size in fish. In addition, carbon stable isotope values (𝛿^13^C) also significantly negatively predicted the Standard Length of *Hoplopteryx* spp., suggesting that other environmental factors, such as primary productivity and/or the burial of organic matter, may also have affected body size.

## Graphical abstract

In this study, we use the extensive fossil record of the extinct Late Cretaceous Trachichthyid fish genus, *Hoplopteryx*, from the British Chalk Group to explore how this family responded to past climate change events. Our study is the first to demonstrate that seawater palaeotemperature, estimated from oxygen stable isotope values, is a significant negative predictor of body size in the species *Hoplopteryx lewesiensis*. Additionally, we show that carbon stable isotope values significantly negatively predict *Hoplopteryx* body size, indicating that other palaeoenvironmental factors also influenced body size within this group of fish.

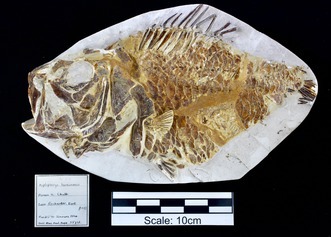

## 1 Introduction

Body size is a fundamental trait that affects many aspects of an animal's life history, ecology, and evolution (Peters 1983). It is also a trait that responds to external, environmental factors, such as temperature, as outlined by the ecogeographical rules of Bergmann (1847) and James (1970), and the temperature–size rule (Atkinson 1994), where warmer waters tend to be inhabited by smaller‐sized species and individuals (Daufresne et al. 2009). This relationship between temperature and body size has been tested in many living fish; for example, van Rijn et al. (2017) showed that the maximum annual sea surface temperature is the best predictor of body size in 74 species.

Based on this relationship, it is predicted that as global temperatures increase with current climate change, many species will become smaller in response (Forster et al. 2012; Millien et al. 2006; Sheridan and Bickford 2011). Fish are not expected to respond to temperature alone but to a range of related factors, such as dissolved oxygen content, pH, and productivity (Mora et al. 2013). For example, as seawater temperature increases, the dissolved oxygen content decreases, and fish growth rate slows down (Pörtner and Knust 2007). By 2050, it is predicted that fish maximum body weight will have decreased by up to 24% (Cheung et al. 2013). There is evidence that larger‐bodied, commercially important species are disproportionately decreasing in size and abundance compared to smaller, non‐commercially important species (Baudron et al. 2014; Genner et al. 2010; Todd et al. 2008). However, fishing is size‐selective, and commercial pressure likely limits the ability of larger, commercially important species to respond to climate change by disproportionately removing reproductive adults (Genner et al. 2010).

Larger body sizes are associated with increased survival against predators in young fish (White et al. 2013) and greater reproductive output and success in mature fish (Barneche et al. 2018; Hixon et al. 2014). Therefore, body size decreases resulting from temperature changes may have negative impacts on fish at individual, population, and ecosystem levels, including reduced fecundity and reduced offspring survival (Ahti et al. 2020). These negative impacts could result in population declines for species that grow and reproduce slowly. Further research is necessary to understand how climate change may affect different fish species, particularly those that are more vulnerable, such as those with slow life histories.

Trachichthyidae, also known as ‘slimeheads’, are a family of commercially exploited fish found in the Atlantic, Pacific, and Indian oceans (Nelson et al. 2016). They typically occupy depths ranging from 100 to 1500 m, depending on species and maturity (Lorance et al. 2002; Madurell and Cartes 2005), where they are sluggish predators feeding on crustaceans, other fish, and squid (Bulman and Koslow 1992; Madurell and Cartes 2005). Trachichthyidae have relatively high metabolic rates for deep‐sea fish, meaning that they grow slowly and inefficiently despite their high food consumption (Bulman and Koslow 1992). They mature late in life (some species do not reach reproductive maturity until 20 years old) and display low fecundity, as they produce a relatively small number of offspring (D'onghia et al. 1998; Mace et al. 1990). They are extremely long‐lived, with individuals of some species documented to reach 50–100 years of age (Lorance et al. 2002; Mace et al. 1990).

It is extremely difficult to observe biological changes in Trachichthyidae over short study periods due to their deep‐sea habitat and slow life history (Clark et al. 2000), and it has not been possible to experimentally test the temperature–size relationship in these fish. Only one species, *Hoplostethus atlanticus* , has been subject to any temperature‐related studies. Using otoliths to estimate growth rates and the Mg/Ca ratio of deep‐sea corals as a temperature proxy, Thresher et al. (2007) inferred a positive relationship between the growth rate of juvenile *H. atlanticus* and temperature over the past 200 years. How temperature may relate to adult body size was not examined.

In this study, we take a different approach and utilise the fossil record as an alternative source of data to test the temperature‐size relationship in the Trachichthyidae. Originating in the Cretaceous (Berg 1958; Patterson 1964), the Trachichthyidae have a long evolutionary history and have experienced many past episodes of global climate change. The fossil record of these events can be viewed as a series of ‘natural experiments’ (Jablonski 2004) that provide an opportunity to test the relationship between palaeotemperature and body size in extinct members of extant clades. This may contribute to better prediction of the responses of living species to current climate change (Millien et al. 2006).

We focused on the extinct trachichthyid genus *Hoplopteryx*, which ranges from the Cenomanian to Campanian stages of the Late Cretaceous (ca. 100.5 to 72.1 million years ago) (Cohen et al. 2013; Patterson 1964). Fossils of *Hoplopteryx* are relatively common in the British Chalk Group of southern England, which was originally deposited as a coccolith ooze on the floor of an epeiric sea no deeper than 200–500 m (Bell et al. 1999). Chalk fossils have been collected for over 200 years, and specimens of *Hoplopteryx* are housed in many United Kingdom (UK) museum collections. Furthermore, the Chalk Group has been the subject of palaeoclimatological studies for more than three decades as it records several significant global warming and cooling events (Jarvis et al. 2006; Jenkyns et al. 1994), and geochemical methods are well established for estimating trends in Chalk Sea water temperature from oxygen stable isotope values (𝛿^18^O; Anderson and Arthur 1983; Jenkyns et al. 1994).

This study aimed to provide the first test of the temperature‐size relationship in the Trachichthyidae by analysing size change in the extinct, Late Cretaceous genus *Hoplopteryx* from the British Chalk Group of southern England. Fossil fish body size data were compared to estimates of seawater palaeotemperature inferred from the 𝛿^18^O of the bulk chalk matrix surrounding individual fossils. To explore other potential palaeoenvironmental influences on body size, we also investigated the relationship between size and carbon stable isotope values (𝛿^13^C).

## 2 Materials and Methods

### 2.1 Materials

Fossilised specimens of *Hoplopteryx* were sourced from the Natural History Museum, London (NHMUK), the British Geological Survey, Keyworth (BGS), the Grant Museum of Zoology, London (GMZ) and the Sedgwick Museum of Earth Sciences, Cambridge (CSM). In total, 250 *Hoplopteryx* specimens were surveyed. Ten specimens labelled *Hoplopteryx superbus* were excluded from this study, as it is uncertain whether this species belongs to *Hoplopteryx* or to *Caproberyx* (Patterson 1964). One further specimen was excluded due to its poor taxonomic resolution, i.e., labelled ‘*Hoplopteryx?*’. Of the remaining specimens, a further 26 were not sufficiently well articulated or preserved to be analysed further, leaving a total dataset of 213 specimens. All specimens were originally collected from inland and coastal exposures of the British Chalk Group of southern England, UK (Figure S1).

### 2.2 Measurements

**FIGURE 1:**
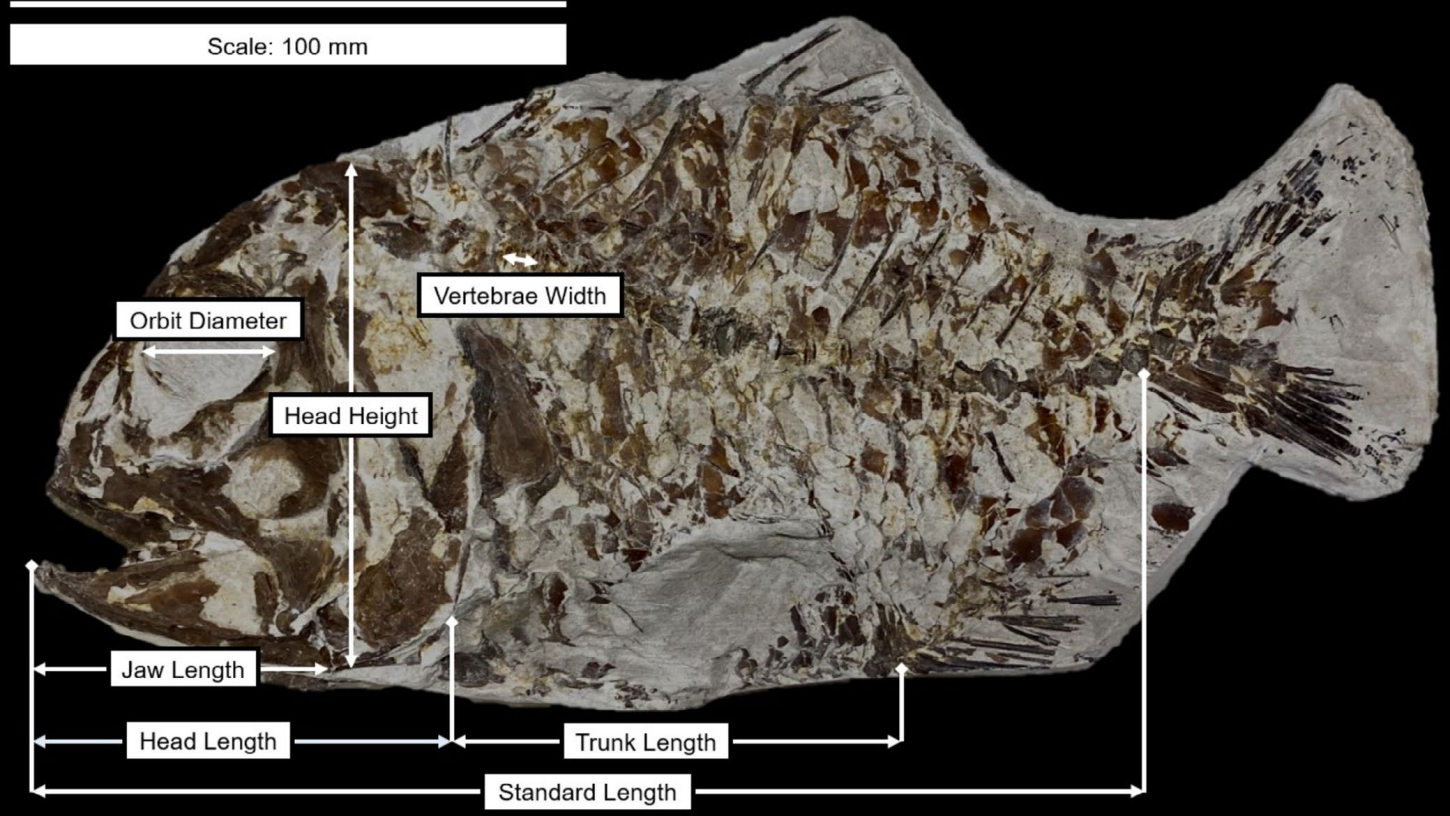
*Hoplopteryx lewesiensis* specimen (NHMUK PV P 51289) labelled with the seven morphological parameters measured in this study (definitions in Table 1).

Seven morphological parameters (Figure 1; Table 1) were measured using Mitutoyo digital callipers accurate to 0.01 mm. All specimens were measured once, and a random subset of 23 were re‐measured to verify internal consistency and reproducibility. All repeats were within ±2% (Table S1). For these repeat‐measured specimens, the mean values between the two measurements were used in all further analyses. Standard Length (SL), measured as the length from the tip of the snout to the base of the tail, was chosen to represent body size in this study. Three fish were preserved with their bodies in a curved or twisted position. In order to measure SL in those specimens, a piece of string was used that was then straightened out and measured with callipers. The process was repeated three times, and a mean SL was calculated.

**TABLE 1:** Definitions of the seven morphological parameters measured in this study.

Measurement	Definition
Head length	Length from the tip of the snout to the anterior of the operculum
Head height	Length from the most dorsal to the most ventral point of the head, perpendicular to head length
Jaw length	Length from the most anterior to the most posterior point of the visible jawbone(s)
Orbit diameter	Length of the eye socket from the most anterior to the most posterior point
Trunk length	Length from the most anterior point behind the head, to the point just before any preserved anal fin rays
Vertebrae width	Length of the most anterior visible vertebra, from its most anterior to most posterior point
Standard length (SL)	Length from the tip of the snout to the base of the tail

### 2.3 Morphometric Analyses

Most fossils (78.4% of the specimen dataset) were incomplete or still retained adhering chalk matrix, which prevented direct measurement of the SL. To include these specimens in our study, morphometric analyses were undertaken to determine whether alternative morphological parameters could provide robust estimates of SL in partial specimens, and, if so, which parameter(s) gave the most precise and accurate estimate. Six skeletal parameters were tested: Head Length, Head Height, Jaw Length, Orbit Diameter, Trunk Length, and Vertebrae Width (Table 1). These parameters were measured in the most complete specimens of *Hoplopteryx* spp. (*n* = 46) and linear regressions between each parameter and SL were generated. The robustness of each morphological parameter for explaining SL was determined by the *R* ^2^, *F*‐statistic, and residual standard error of each linear regression (Engelman 2023; Grouard et al. 2019). The outputs of linear regressions, which included the best‐performing parameters as predictors, were then utilised to estimate SL in partial specimens (i.e., Standard Length ~ Best‐Performing Parameter). The outputs of these regressions were also used to estimate SL in complete specimens to compare with their measured SL.

### 2.4 Carbon and Oxygen Stable Isotope Analysis

Samples of chalk matrix were taken directly adjacent to the preserved remains of 54 NHMUK *Hoplopteryx* specimens, which were selected on completeness and to encompass as large a range of recorded body size as possible. Bulk chalk matrix was weighed (650–750 μg each) and transferred to a 12 mL Labco vial that was then screw‐capped with butyl rubber septa and flushed with ultra‐pure helium. Samples were reacted with 100 μL phosphoric acid (density 1.92 g mL^−1^).

Stable isotope analysis was carried out on a DELTA V Advantage continuous flow isotope ratio mass spectrometer (IRMS) linked to a GasBench II via a ConFlo IV with a GC PAL autosampler (Thermo Fisher Scientific, Germany) at the Bloomsbury Environmental Stable Isotope Facility at University College London in London, UK. IRMS linearity was always < 0.06‰ volt^−1^ for a defined span of carbon dioxide (CO_2_) voltage, and the standard deviation of values of δ^13^C and δ^18^O from ten peaks of CO_2_ working gas was always < 0.06‰.

Sample CO_2_ evolved from acidification was purged from vials through a double‐hole sampling needle into a helium carrier. After drying, the CO_2_ was separated from residual gases by a chromatographic column (PoraPLOT Q) at 70°C. Repetitive loop injection allowed for the transfer of ten headspace CO_2_ samples per vial. Values of 𝛿^13^C and 𝛿^18^O were derived relative to the CO_2_ working gas introduced directly into the IRMS and were corrected for ^17^O (Santrock et al. 1985) by Isodat software (version 3.0.95.23; Thermo Fisher Scientific, Germany). Stable isotope ratios, expressed as delta values (δ) in per mil units (‰; Brand et al. 2014), represent the ratio of heavy to light isotopes within a sample (R_sample_) relative to the ratio in an international standard (R_standard_): δ = (R_sample_/R_standard_)−1 (Coplen 2011). Sample CO_2_ peaks number two to ten (excluding sample peak one) were used to calculate mean isotopic delta values.

All values of δ^13^C and δ^18^O are reported relative to the international reference standard Vienna Pee Dee Belemnite (VPDB). International Atomic Energy Agency (IAEA) reference materials (RMs) (Tables S2 and S3) were analysed in duplicate at the start and end of each sample batch. A calcium carbonate laboratory standard was analysed at regular intervals throughout each sample batch. Isotopic values from RMs and laboratory standards were used to assess drift and linearity. No drift or linearity corrections were applied. Values were checked for outliers. No oxygen‐isotope acid fractionation factor was applied because the carbonate present in the samples was expected to be calcite. The acid fractionation factor would be identical for samples and reference materials, and its value would therefore cancel out (Kim et al. 2015). Data from IAEA‐603 and IAEA‐610 were used to scale‐correct all delta values (Paul et al. 2007). Accuracy and precision data from the analysis of RMs and laboratory standard calcium carbonate are reported in Tables S2 and S3.

### 2.5 Palaeotemperature Reconstruction

The Upper Cretaceous Chalk of the UK and northwest Europe formed from carbonate ooze which originally accumulated on the seafloor as marine snow and is composed primarily of the microscopic fossil skeletons of calcareous nannoplankton, mainly coccolithophores (nannofossils), with minor components from other biotic and abiotic sources (Fabricius 2007; Püttmann and Mutterlose 2021). Therefore, the oxygen and carbon isotope compositions of the bulk chalk sampled directly adjacent to the fossil fish can provide suitable insight into the environmental conditions in the upper parts of the water column during the life of the fish, given the temporal resolution of the fossil record, as experienced by the calcareous nannoplankton preserved as fossils within it (Wefer and Berger 1991).

Following the approach of Jenkyns et al. (1994), the relative seawater palaeotemperature of each chalk sample was estimated using the equation:

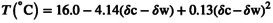

in which 𝛿c is the measured oxygen isotope composition of the bulk chalk matrix and 𝛿w is the oxygen isotope composition of seawater (Anderson and Arthur 1983). The oxygen isotope composition of Chalk Sea seawater is unknown. Therefore, in common with many other palaeoclimate studies, a seawater δ^18^O_SMOW (Standard Mean Ocean Water)_ of −1‰ was used, based on the assumption that this records the average value for an ice‐free, greenhouse Late Cretaceous world (Wilkin 2021).

### 2.6 Statistical Analyses

To determine the influence of 𝛿^18^O‐derived seawater temperature and 𝛿^13^C on SL, a multiple linear regression model was employed, specified as Standard Length ~ 𝛿^18^O‐derived seawater temperature + 𝛿^13^C. Limited stratigraphic and locality data were available for the specimens sampled for geochemical analyses, so these variables were not incorporated into the model. Separate models were employed at the genus level (i.e., the entire dataset) and at the species level, where sufficient specimens (minimum *n* = 20) were present.

Each model was assessed against the assumptions of multiple linear regression to verify the robustness of our analyses. Scatterplots indicated that both 𝛿^18^O‐derived seawater temperature and 𝛿^13^C demonstrated the expected linear relationship with SL. Diagnostic plots demonstrated that model residuals were suitably normally distributed and homoscedastic. Collinearity was not present in any model, as indicated by suitably low Variance Inflation Factors.

## 3 Results

### 3.1 Morphometric Analyses

**FIGURE 2:**
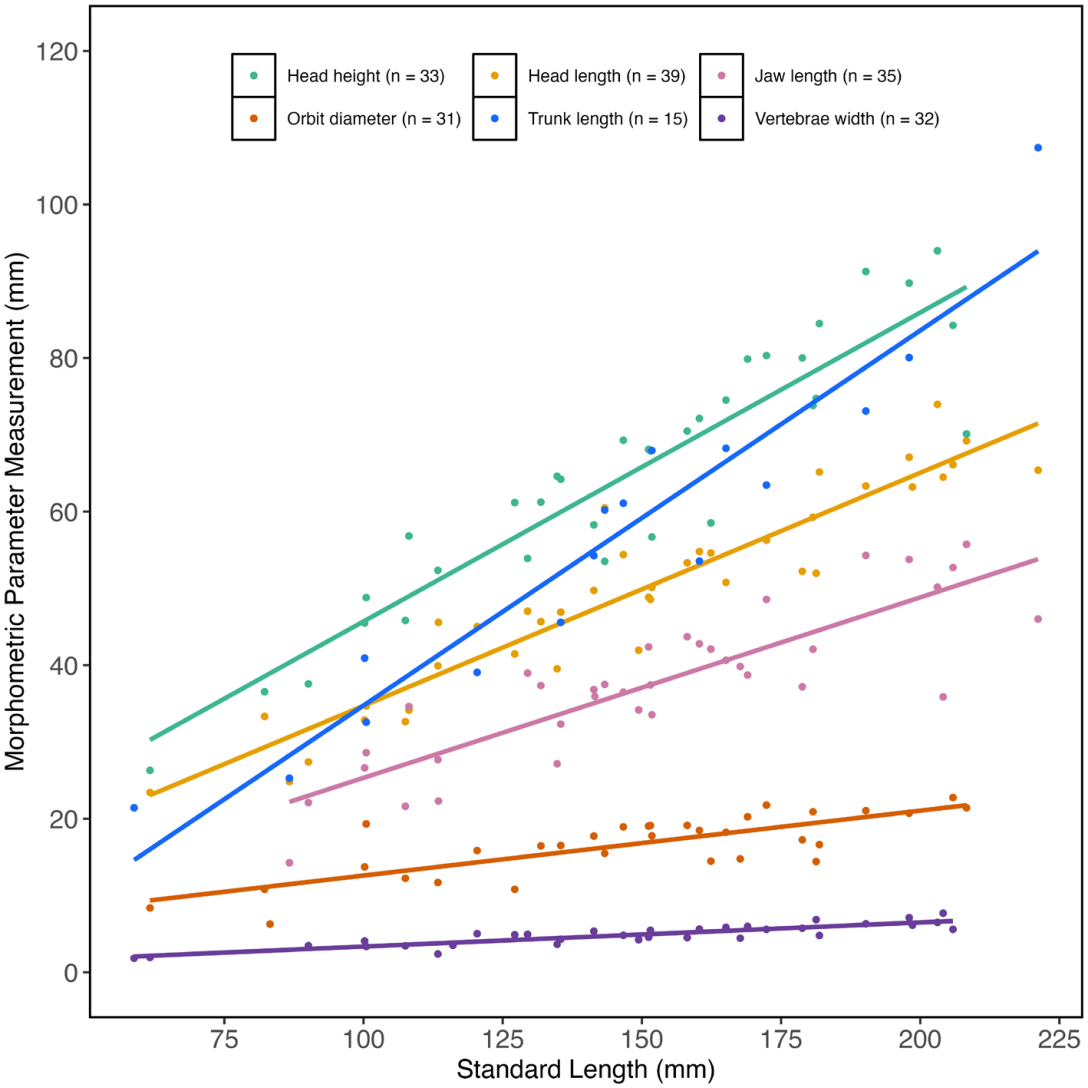
The relationship between Standard Length (SL) and each morphometric parameter in *Hoplopteryx* spp. Each dot represents one specimen. Colours represent different morphological parameters, as shown in the key. A regression line (y~x) has been fitted to each parameter dataset. Sample sizes are shown on the plot.

Of the 213 specimens included in this study, only 46 had directly measurable SLs, which ranged from 58.8 mm to 221.2 mm (Table S4). Morphometric analyses of those 46 specimens showed that each of the six morphological parameters tested increased with SL (Figure 2). Following regression analyses, Orbit Diameter, Vertebrae Width, and Jaw Length were discounted as predictors of SL as the *R* ^2^ was lower than 80%, the *F*‐statistics were relatively low, and the residual error was relatively high (Table 2), indicating that these parameters poorly explained the variability in SL. Although Trunk Length *R* ^2^ was higher than 80%, with a relatively high *F*‐statistic and relatively low residual error, the sample size was much smaller than that of the other parameters (*n* < 20). Therefore, Trunk Length was also discounted as a predictor of SL.

**TABLE 2:** Results of regression analyses of each morphological parameter.

Predictor	*n*	*R* ^2^	*F*	Residual standard error
Head length	39	0.89	294.4	13.69 on 38 df
Head height	33	0.85	185.8	14.53 on 32 df
Trunk length	15	0.91	142.3	13.28 on 14 df
Vertebrae width	32	0.78	110.3	18.94 on 31 df
Jaw length	35	0.74	96.35	18.54 on 34 df
Orbit diameter	31	0.61	46.23	23.70 on 30 df

Head Length and Head Height demonstrated the highest *R* ^2^ values, largest *F*‐statistics, and the lowest residual errors, indicating that these two parameters best explain the variability in SL (Table 2). Therefore, estimates of SL for incomplete specimens were generated using regression models selected based on the availability of measurements for these parameters in the specimens. When both Head Height and Head Length were available on a specimen, a multiple linear regression model was used (Standard Length ~ Head Length + Head Height). When only Head Length or Head Height were available, single variable linear regression models were used accordingly (Standard Length ~ Head Length or Standard Length ~ Head Height).

### 3.2 Relationships Between Body Size and Palaeoenvironmental Proxies

Of the 54 samples analysed geochemically, 26 were associated with specimens of fish where SL was directly measured, and 28 where SL was estimated from direct measurements of Head Length, Head Height, or both. 47 samples were from specimens of *H. lewesiensis*, with the remaining 7 either unassigned to species or assigned to other species.

At the genus level, the overall model was statistically significant (Adjusted *R* ^2^ = 0.07, *F*(2, 51) = 3.13, *p* = 0.05). In *H. lewesiensis* only, the overall model was also statistically significant (Adjusted *R* ^2^ = 0.28, *F*(2, 44) = 10.16, *p* = < 0.001). This suggests the models explain a suitable portion of the variance in SL.

### 3.3 Size and δ ^18^O‐Derived Seawater Temperature

**FIGURE 3:**
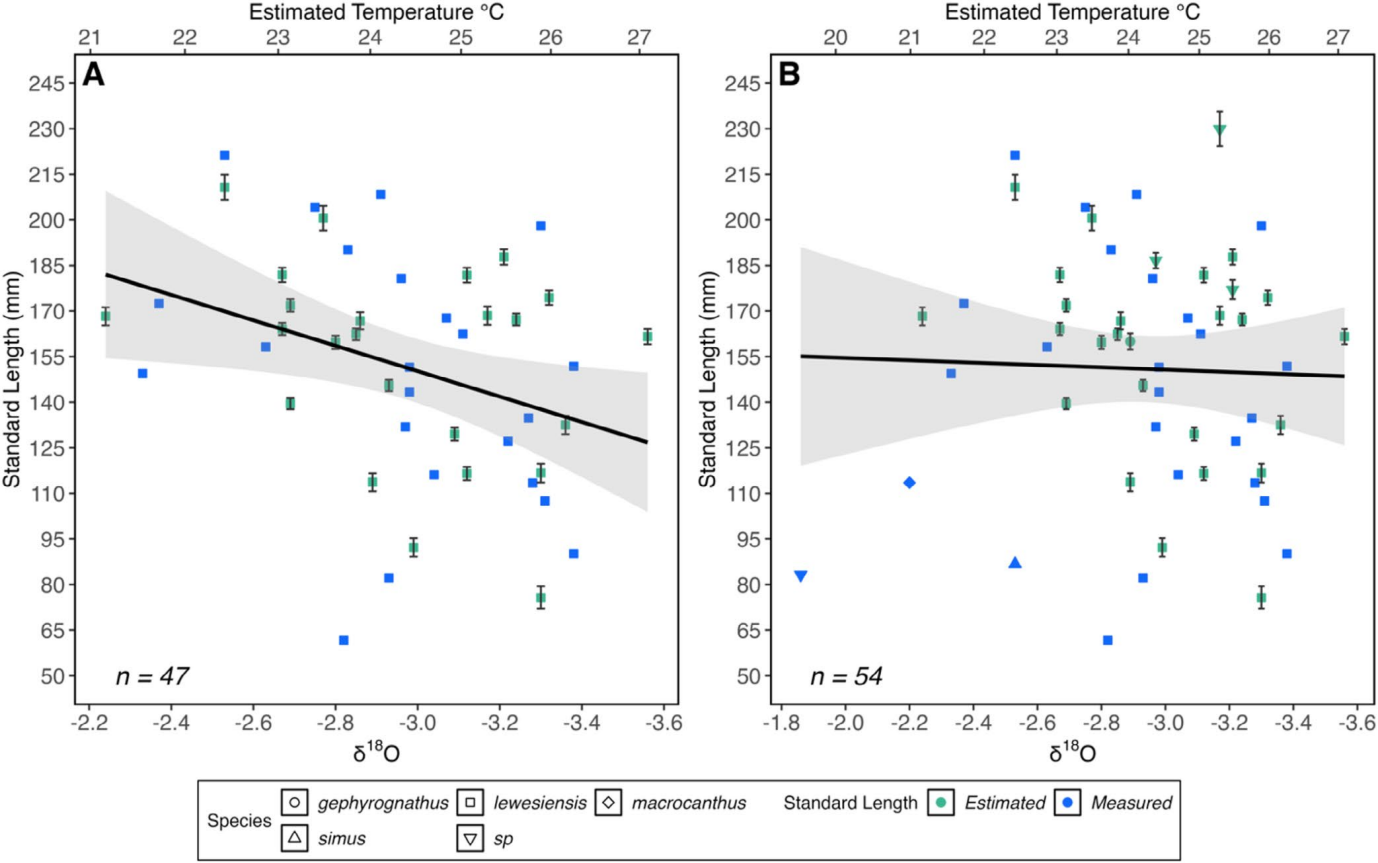
The relationship between 𝛿^18^O‐derived seawater temperature estimates and Standard Length (SL) in (A) *Hoplopteryx lewesiensis* and (B) *Hoplopteryx* spp. Estimated points show the SL generated from a suitable linear regression. Measured points either show the true SL measurement or a mean of two repeats. The error bars on estimated SL points show the standard error of the estimate. No error bars are shown on measured SL points as the smallest and largest values of repeat measurements were smaller than the size of the symbol. Sample sizes are shown on each plot. A regression line (y~x) is added to each plot, and the grey‐shaded areas represent a 95% confidence interval.

Measured 𝛿^18^O values ranged from −1.9‰ to −3.6‰, which equated to seawater temperature estimates of 19.5°C–27.1°C, respectively (Table 3). In *H. lewesiensis* only (*n* = 47), 𝛿^18^O‐derived seawater temperature was a significant negative predictor of SL (β = −7.70, *p* = 0.03), indicating that sizes are smaller at higher estimated temperatures (Figure 3A). There were insufficient specimens to test this relationship in other *Hoplopteryx* species separately. However, in the whole *Hoplopteryx* spp. dataset (*n* = 54), estimated seawater temperature was not a significant predictor of SL (β = −0.14, *p* = 0.97) (Figure 3B).

**TABLE 3:** Geochemical and Standard Length (SL) data collected from 54 *Hoplopteryx* spp. specimens at the Natural History Museum, UK.

NHMUK registration no.	Species	δ^18^O	Temperature (°C)	δ^13^C	SL	Minimum SL	Maximum SL
PV OR 36917a	*lewesiensis*	−2.82	23.7	2.29	*61.6*	61.4	61.9
PV P 5423	*lewesiensis*	−3.3	25.9	3.02	**75.7**	72.0	79.4
PV OR 25827	*lewesiensis*	−2.93	24.2	2.09	*82.2*	81.4	83.1
PV OR 41993	sp	−1.86	19.5	2.02	83.2	—	—
PV P 10222	*simus*	−2.53	22.4	1.62	*86.7*	85.9	87.5
PV OR 4012	*lewesiensis*	−3.38	26.3	2.32	90.1	—	—
PV P 6464	*lewesiensis*	−2.99	24.5	2.99	**92.2**	89.1	95.2
PV OR 4008	*lewesiensis*	−3.31	25.9	2.41	*107.5*	107.5	107.6
PV OR 4026	*lewesiensis*	−3.28	25.8	2.4	*113.4*	111.9	114.8
PV OR 33230	*macrocanthus*	−2.2	21	1.91	113.5	—	—
PV OR 49038	*lewesiensis*	−2.89	24	2.24	**113.7**	110.7	116.6
PV P 1948(d)	*lewesiensis*	−3.04	24.7	3.01	116.1	—	—
PV P 1948(a) (1)	*lewesiensis*	−3.12	25.1	1.93	**116.5**	114.3	118.7
PV OR 25912	*lewesiensis*	−3.3	25.9	1.38	**116.7**	113.6	119.8
PV OR 4011	*lewesiensis*	−3.22	25.5	2.28	127.2	—	—
PV OR 25863	*lewesiensis*	−3.09	24.9	2.39	**129.5**	127.3	131.6
PV OR 49041(1)	*lewesiensis*	−2.97	24.4	1.58	131.9	—	—
PV OR 49888	*lewesiensis*	−3.36	26.2	2.88	**132.5**	129.5	135.5
PV OR 49867(1)	*lewesiensis*	−3.27	25.7	1.92	134.8	—	—
PV P 5420	*lewesiensis*	−2.69	23.1	3.14	**139.5**	137.7	141.3
PV P 4842	*lewesiensis*	−2.98	24.4	2.21	143.3	—	—
PV OR 4019	*lewesiensis*	−2.93	24.2	1.53	**145.5**	143.6	147.4
PV OR 4014	*lewesiensis*	−2.33	21.5	1.94	*149.4*	148.8	150.1
PV OR 4016	*lewesiensis*	−2.98	24.4	2.83	*151.5*	149.1	154.0
PV OR 49870	*lewesiensis*	−3.38	26.3	2.35	151.8	—	—
PV OR 25781	*lewesiensis*	−2.63	22.9	3.01	*158.2*	157.7	158.6
PV OR 4106	*lewesiensis*	−2.8	23.6	2.12	**159.7**	157.5	161.9
PV OR 41104	*gephyrognathus*	−2.89	24	4.24	**160.0**	157.3	162.6
PV P 7189	*lewesiensis*	−3.56	27.1	1.92	**161.6**	159.1	164.0
PV OR 25841	*lewesiensis*	−3.11	25	1.91	162.4	—	—
PV P 5690	*lewesiensis*	−2.85	23.8	2	**162.4**	160.4	164.3
PV OR 41105(a)	*lewesiensis*	−2.67	23	2.03	**164.1**	162.1	166.1
PV OR 4109	*lewesiensis*	−2.86	23.9	1.3	**166.8**	163.9	169.6
PV P 5692	*lewesiensis*	−3.24	25.6	1.71	**167.2**	165.3	169.2
PV P 73798	*lewesiensis*	−3.07	24.8	2.98	167.7	—	—
PV OR 4015	*lewesiensis*	−2.24	21.2	1.93	**168.3**	165.3	171.2
PV P 5693	*lewesiensis*	−3.17	25.3	1.7	**168.5**	165.5	171.5
PV P 1948(b)	*lewesiensis*	−2.69	23.1	1.75	**171.8**	169.8	173.9
PV OR 79	*lewesiensis*	−2.37	21.7	1.98	*172.4*	171.4	173.4
PV OR 4021a	*lewesiensis*	−3.32	26	1.96	**174.4**	172.0	176.7
PV OR 28392	sp	−3.21	25.5	1.41	**177.0**	173.9	180.2
PV P 5694	*lewesiensis*	−2.96	24.3	1.92	180.7	—	—
PV P 5688	*lewesiensis*	−3.12	25.1	1.71	**181.8**	179.4	184.2
PV OR 49037	*lewesiensis*	−2.67	23	2.02	**181.9**	179.5	184.3
PV P 4297	sp	−2.97	24.4	1.98	**186.6**	184.0	189.2
PV P 5689	*lewesiensis*	−3.21	25.5	1.73	**187.8**	185.3	190.3
PV P 51289	*lewesiensis*	−2.83	23.8	1.96	190.2	—	—
PV P 5687	*lewesiensis*	−3.3	25.9	1.62	*198.0*	196.09	199.95
PV OR 49862	*lewesiensis*	−2.77	23.5	1.96	**200.6**	196.4	204.7
PV P 9909	*lewesiensis*	−2.75	23.4	1.27	204.1	—	—
PV OR 35712	*lewesiensis*	−2.91	24.1	2.1	*208.3*	208.2	208.5
PV OR 49863	*lewesiensis*	−2.53	22.4	1.47	**210.7**	206.5	214.9
PV OR 49043	*lewesiensis*	−2.53	22.4	1.63	*221.2*	220.6	221.8
PV OR 4239	sp	−3.17	25.3	1.93	**229.9**	224.3	235.6

*Note:* Estimated SL values are in bold. Italicised SL is the mean of two measurements. Minimum and maximum estimated or measured SL values are listed where applicable.

### 3.4 Size and δ ^13^C

**FIGURE 4:**
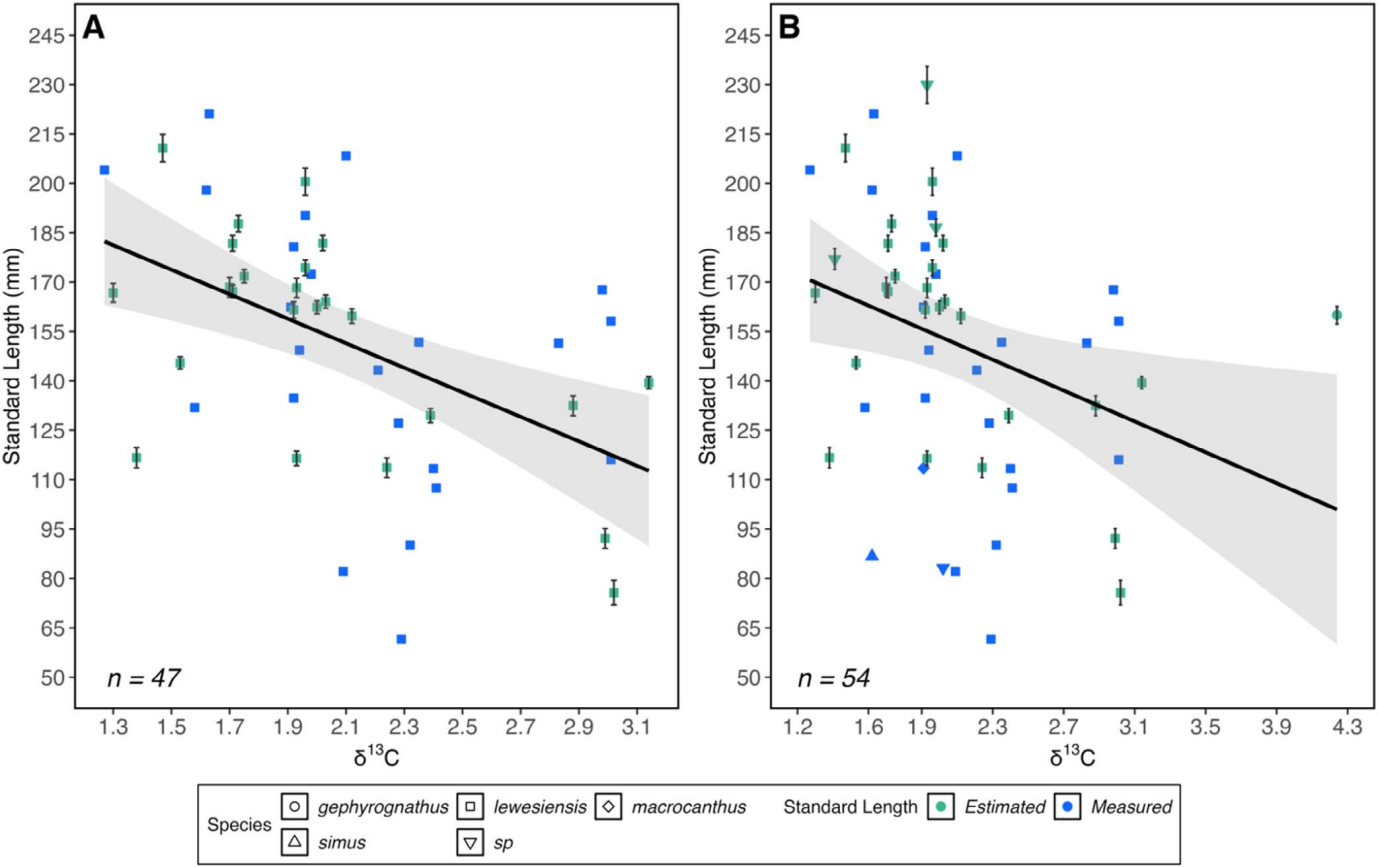
The relationship between 𝛿^13^C and Standard Length (SL) in (A) *Hoplopteryx lewesiensis* and (B) *Hoplopteryx* spp. Estimated points show the SL generated from a suitable linear regression. Measured points either show the true SL measurement or a mean of two repeats. The error bars on estimated SL points show the standard error of the estimate. No error bars are shown on measured SL points as the smallest and largest values of repeat measurements were smaller than the size of the symbol. Sample sizes are shown on each plot. A regression line (y~x) is added to each plot, and the grey‐shaded areas represent a 95% confidence interval.

Measured 𝛿^13^C values ranged from 1.3‰ to 4.2‰ across the 54 samples (Table 3). In both *H. lewesiensis* and *Hoplopteryx* spp., 𝛿^13^C was a significant negative predictor of SL (β = −34.44, *p* = < 0.001 and β = −23.42, *p* = 0.02, respectively) (Figure 4). Notably, there was no significant relationship between 𝛿^13^C and 𝛿^18^O in the chalk matrix samples (*R* ^2^ = < 0.01) (Figure S2), suggesting the two palaeoenvironmental proxies are independent of each other. Furthermore, the lack of correlation between carbon and oxygen isotope values is an indicator of weak pore fluid–rock interaction and therefore minimal diagenetic alteration to isotopic signatures (Huber et al. 2024).

## 4 Discussion

### 4.1 Body Size and Seawater Temperature

By combining morphometric measurements of fossil specimens with geochemical analysis of their surrounding chalk matrix, our study provides the first test of the temperature‐size relationship in an extinct member of the extant and commercially important deep‐sea fish family Trachichthyidae. When 𝛿^18^O values of the chalk matrix taken directly adjacent to the preserved remains of individual specimens were converted to estimates of seawater temperature, following a well‐established approach and using standard assumptions, our results show that the estimated seawater temperature was a significant negative predictor of Standard Length of individuals of the species *Hoplopteryx lewesiensis*, which is the most common trachichthyid species from the Upper Cretaceous British Chalk Group, i.e., *H. lewesiensis* body size decreased with increasing seawater palaeotemperature.

These results are consistent with studies of similarly sized extant fish, which show that higher temperatures have led to smaller body sizes (Audzijonyte et al. 2020). They provide further support for the prediction that marine animals in general, and fish in particular, will decline in body size with current and future ocean warming owing to a reduction in dissolved oxygen concentrations (e.g., Sheridan and Bickford 2011; Cheung et al. 2013). Although it is possible that the Trachichthyidae may have evolved greater resilience to temperature change since the Late Cretaceous, given the limited data available for living slimeheads (Clark et al. 2000; Thresher et al. 2007), our results suggest that extant members of Trachichthyidae may be expected to decrease in size with current climate warming.

Estimated seawater temperature was not a significant predictor of body size in our genus‐level analysis, which we attribute to the confounding effect of intraspecific variation in body size in the different *Hoplopteryx* species (Patterson 1964). Furthermore, a number of specimens within our genus‐level database were unable to be assigned to individual species, and it is possible that they may represent juveniles rather than adults (see discussion below). For those specimens that were assigned to other species (e.g., to *H. simus* , *H. macrocanthus*), there were insufficient numbers to undertake robust species‐level statistical analyses.

### 4.2 Body Size and δ ^13^C

Across the *Hoplopteryx* genus and for the species *H. lewesiensis* alone, the δ^13^C value of the chalk matrix surrounding individual specimens was found to be a significant negative predictor of Standard Length, with the smallest fish being associated with the most positive δ^13^C values. The interpretation of these results is not straightforward because there are many interacting biotic and abiotic factors that can affect the 𝛿^13^C signature of marine carbonates such as chalk. The primary controls on 𝛿^13^C in chalk are widely considered to be primary productivity and burial of organic matter in seafloor sediments (Jarvis et al. 2002, 2006; Mitchell et al. 1996). Carbon isotope fractionation during marine photosynthesis leads to organic matter enriched in ^12^C and the remaining carbon pool in the surrounding water, from which marine organisms precipitate their skeletal carbonates and, therefore, become relatively enriched in ^13^C (Jarvis et al. 2002). The burial of this ^13^C‐depleted organic matter in seafloor sediments further enhances this process by removing relatively more ^12^C from the system. Thus, during times of enhanced productivity and/or greater burial of organic matter, the 𝛿^13^C of marine carbonates is expected to be more positive (e.g., Mitchell et al. 1996). Our results, therefore, indicate that *Hoplopteryx* became smaller with an increase in productivity and/or burial of organic matter.

Interpreting these results in terms of changes in productivity is problematic. Although we have no direct evidence of the diet of *Hoplopteryx*, it might be expected that greater primary productivity would have increased available food supply at higher trophic levels, which in turn would lead to an increase in *Hoplopteryx* body size (e.g., Mora et al. 2013 and references therein). Unless, perhaps, *Hoplopteryx* was adapted to more oligotrophic conditions, which have been inferred for the offshore environments of the UK chalk seas (Püttmann and Mutterlose 2021), and higher productivity caused a negative impact on body size. Our results are easier to interpret, however, if burial of organic matter is the most significant factor controlling the 𝛿^13^C of the bulk chalk. Increasing organic matter burial could be achieved by increasing the area of seafloor available, for example, at times of higher global sea level (Mitchell et al. 1996), or by reducing the dissolved oxygen content at or near the seafloor, enhancing preservation potential (Jarvis et al. 2002). The latter could explain why *Hoplopteryx* size decreases as 𝛿^13^C increases, as reduced oxygen availability is known to limit fish growth and size (e.g., Cheung et al. 2013; Pörtner and Knust 2007; Sheridan and Bickford 2011).

### 4.3 Ontogeny and Sexual Dimorphism

Standard Length increases through ontogeny, and in some fish, there is also a substantial size difference between the two sexes. It is, unfortunately, often impossible to determine either the sex or ontogenetic stage of a fossilised individual, unless ontogenetic differences or sexual dimorphism are clearly recorded in the preserved skeletal tissues. Here we consider the extent to which either factor could have biased these results.

In extant Trachichthyidae, there is evidence of sexual dimorphism in some species, but not all (D'onghia et al. 1998; Dunn and Forman 2011; Elliott et al. 1995; Shimizu 1977). Even in species where it may occur, the size difference between sexes is relatively small (D'onghia et al. 1998; Elliott et al. 1995; Tracey 1999). Determining the sex of a fossil fish from partial skeletal remains is challenging at best and, in common with nearly all palaeontological studies, we were not able to do this for our specimens. We note, however, that the size distribution of our dataset is unimodal (Figures S3 and S4), and so even if *Hoplopteryx* is shown to have size‐related sexual dimorphism, it is unlikely to have biased our data or caused the significant correlations between Standard Length and chalk 𝛿^18^O values/seawater temperature or chalk 𝛿^13^C values.

The possible impact of ontogeny on our results is more difficult to determine, as methods to determine ontogeny in fish from skeletal remains are limited. Biological age determination using otoliths is the most reliable method of age determination in fishes (Khan et al. 2013). However, otoliths have not been reported from any British Chalk Group fish taxon, probably owing to early diagenetic loss of aragonite (Friedman et al. 2016), so we could not use this technique. Instead, we attempted to assess the biological age of individual specimens by counting the circuli and annuli preserved in their scales. Unfortunately, very few individuals (*n* = 11) preserved visible, measurable circuli, and therefore the correlation between size and age was inconclusive. An alternative approach may be to use computed tomography to image the scales (Thomson and McCune 1984), but this was not possible during our study. Modern studies have shown that Trachichthyidae mature at anywhere between 42% and 73% of their asymptotic length, depending on species (D'onghia et al. 1998; Mace et al. 1990). We do not know what this value would be in *Hoplopteryx*. In the absence of ontogenetic data, we explored removing individuals with sizes less than 50% of the maximum size in our dataset from each 1°C temperature band and re‐running analyses on the remaining 50% of individuals. When doing so, in all cases, there was negligible change in the direction and no change in the significance of the relationships (Figures S5 and S6), and we therefore consider our conclusions robust.

## 5 Conclusion

Our study is the first to demonstrate a significant relationship between seawater palaeotemperature and the body size of an extinct species of Trachichthyidae. We show that estimated seawater temperature, inferred from oxygen isotope (𝛿^18^O) analysis, is a significant negative predictor of body size in the Late Cretaceous species *Hoplopteryx lewesiensis* from the British Chalk Group. Additionally, the carbon isotope value (𝛿^13^C) of the chalk surrounding specimens was a significant negative predictor of *Hoplopteryx* spp. body size, indicating that other abiotic factors also influenced body size in this group of fish through the Late Cretaceous. Our study demonstrates the importance of utilising the fossil record as an alternative source of data to test the temperature‐size relationship in marine animals, such as deep‐sea fish.

## Author Contributions

**Chloe V. Griffiths:** conceptualisation (supporting), data curation (supporting), formal analysis (lead), investigation (lead), methodology (equal), writing – original draft (lead), writing – review and editing (equal). **James D. Witts:** conceptualization (supporting), methodology (equal), writing – review and editing (equal). **Julie C. S. Brown:** conceptualization (supporting), formal analysis (supporting), investigation (supporting), methodology (equal), writing – review and editing (equal). **Emma L. Bernard:** conceptualization (supporting), data curation (lead), methodology (equal), writing – review and editing (equal). **Richard J. Twitchett:** conceptualization (lead), funding acquisition (lead), methodology (equal), writing – review and editing (equal).

## Conflicts of Interest

The authors declare no conflicts of interest.

## Supporting information


**Appendix S1:** ece372026‐sup‐0001‐AppendixS1.docx.

## Data Availability

The authors confirm that the data supporting the findings of this study are available within the article and its Supporting Information. The data are also available from the NHMUK data portal (https://doi.org/10.5519/jda3b5uh).
