# Supplementary material for: Slimehead Size Through Time: Testing the Temperature–Size Relationship in Late Cretaceous Trachichthyidae
Source: Ecol Evol. 2025 Sep 25;15(10):e72026. doi: 10.1002/ece3.72026 (PMC12461127; doi:10.1002/ece3.72026)
Supplement: Supplementary file 1 — Appendix S1: ece372026‐sup‐0001‐AppendixS1.docx. [file ECE3-15-e72026-s001.docx]

# **SUPPLEMENTARY FIGURES**

Figure S1: Map of the Southeast of England, UK, showing the localities of 32 *Hoplopteryx* spp. that were sampled for geochemical analyses with detail at town/city level or better. The chalk outcrop is shaded dark grey. The bubble size represents the number of specimens at each locality. The bubble colour indicates whether a detailed locality (i.e. a particular pit) was noted on the specimen label.


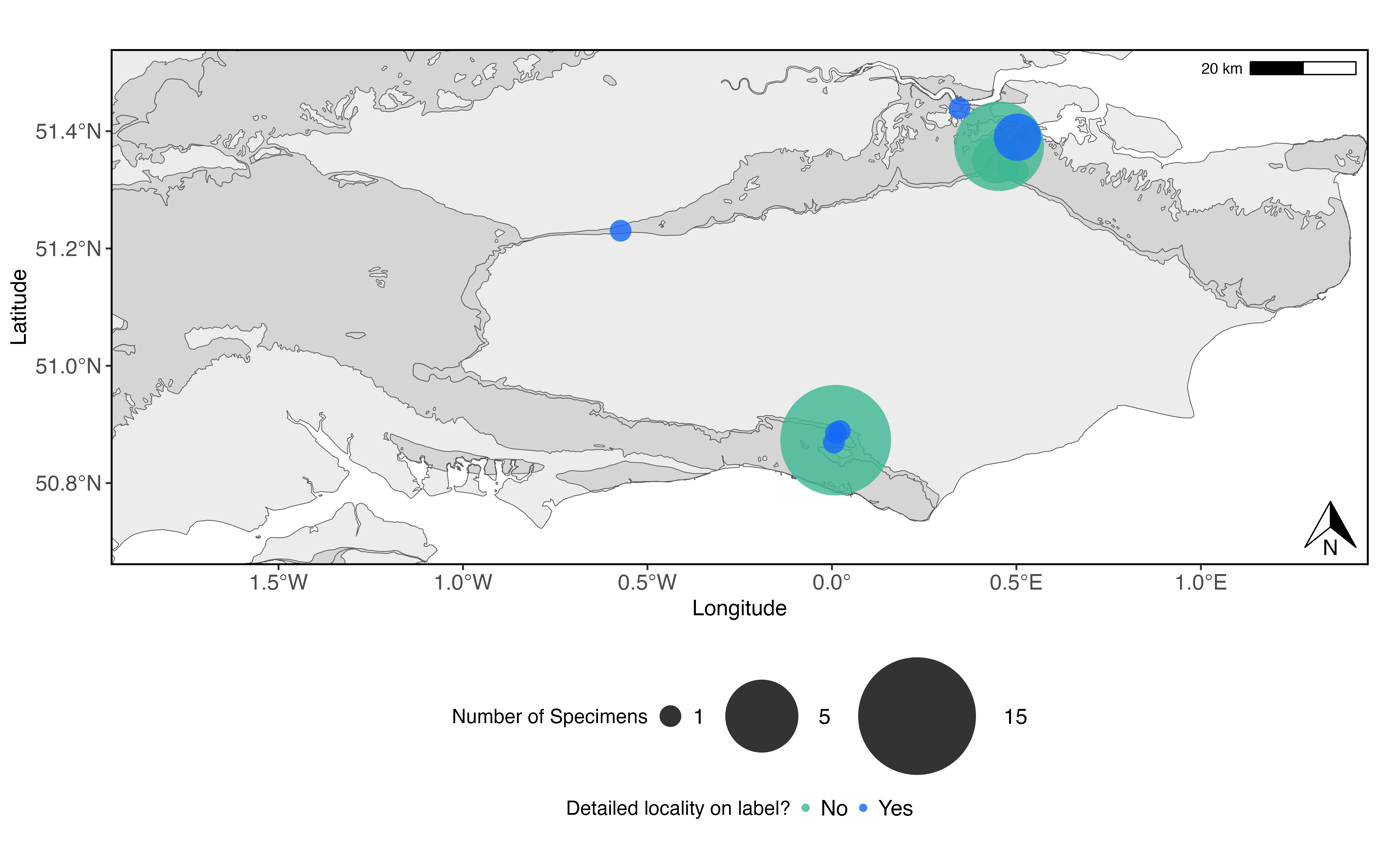


Figure S2: The relationship between 𝛿^18^O and 𝛿^13^C in the bulk chalk matrix of the specimens in this study. A regression line (y~x) is added to the plot and the grey-shaded area represents a 95% confidence interval. The equation of the regression line and *R*^2^ of the correlation are shown on the plot.

*
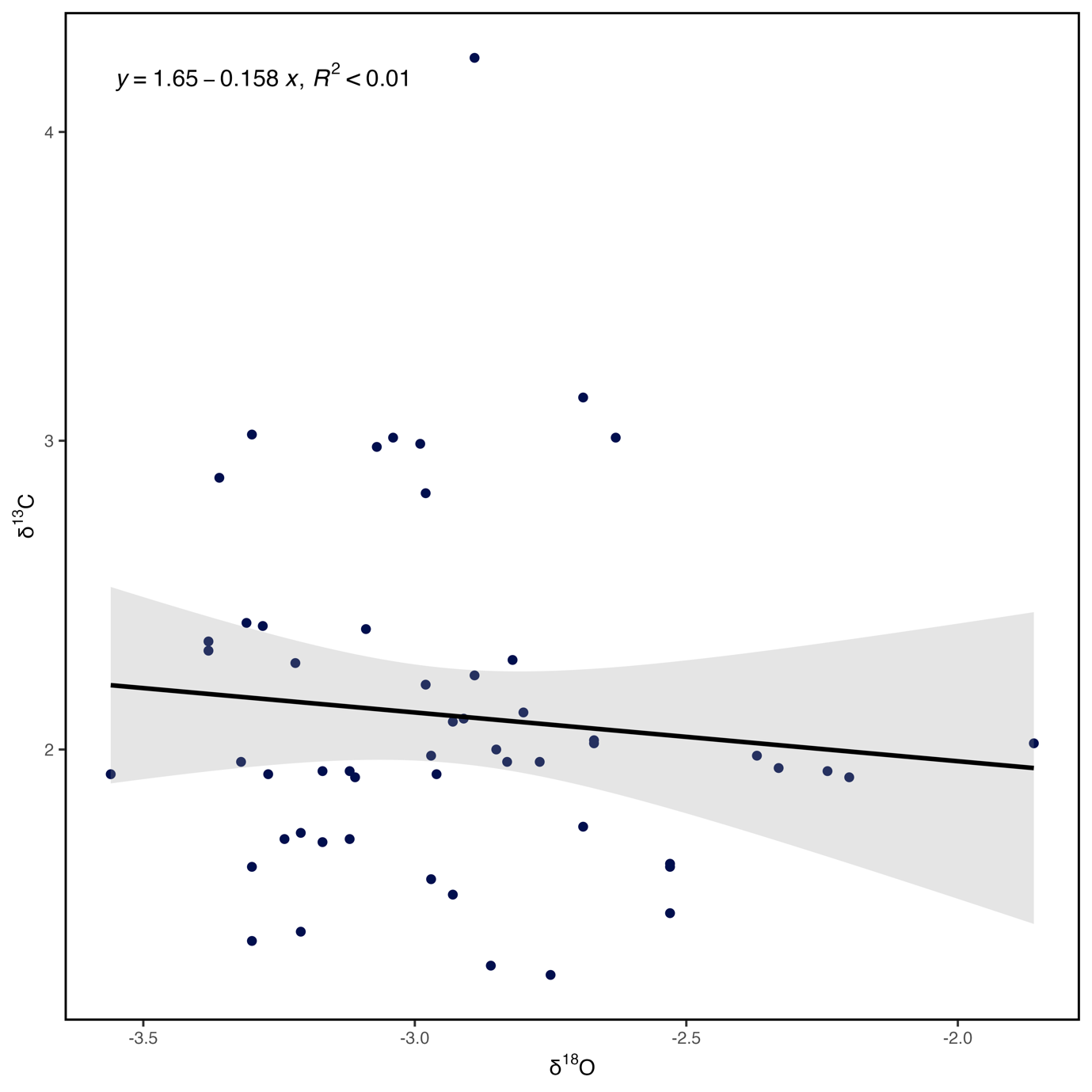
*

Figure S3: Standard Length (SL) distribution of *Hoplopteryx* spp. (*n* = 135). *(*A) SL was measured directly in 46 specimens and estimated in 89. *(*B) Of the 135 specimens, 54 were sampled for geochemistry.


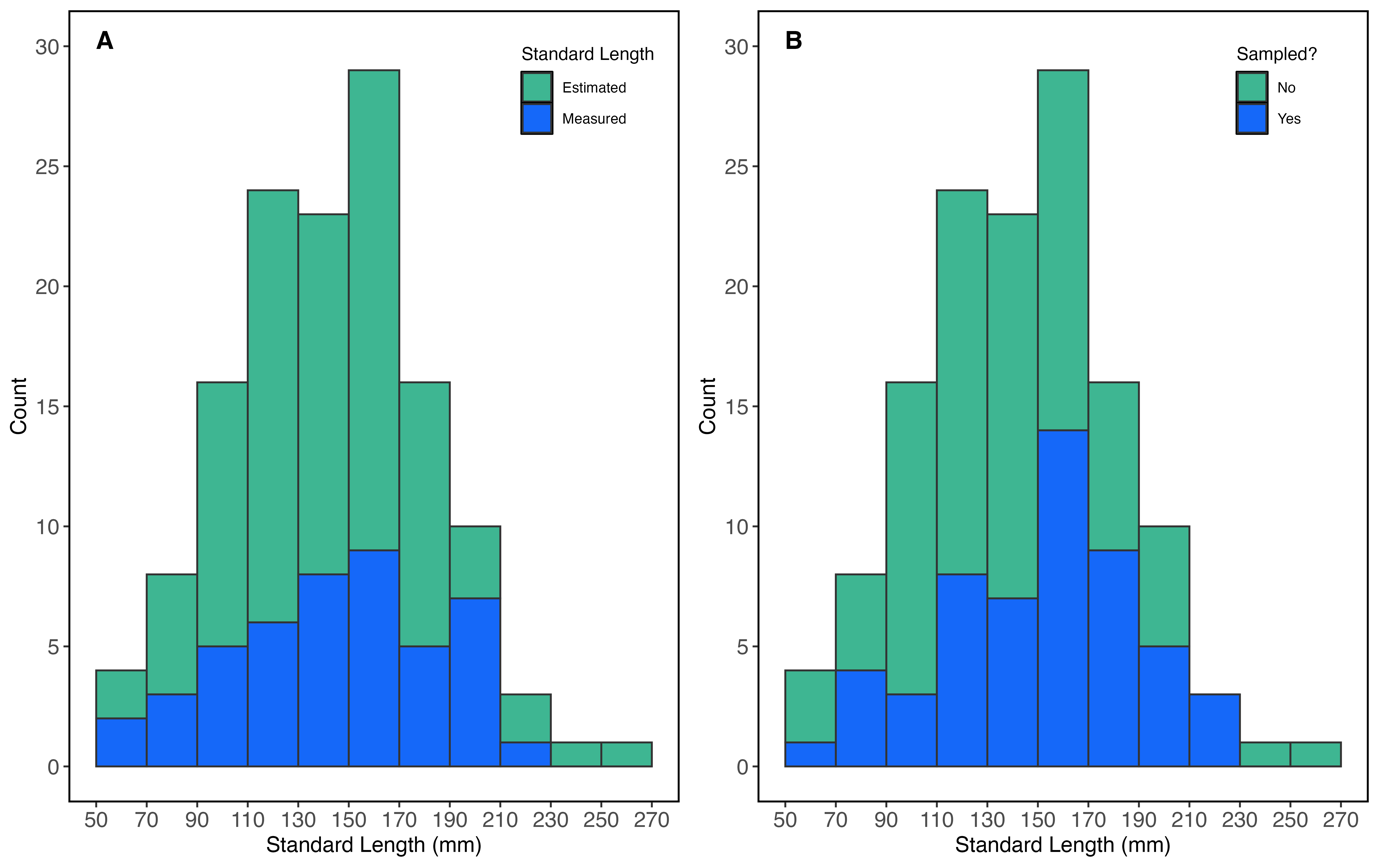


Figure S4: Standard Length (SL) distribution of Hoplopteryx lewesiensis (n = 103). (A) SL was measured directly in 40 specimens and estimated in 63. (B) Of the 103 specimens, 47 were sampled for geochemistry.


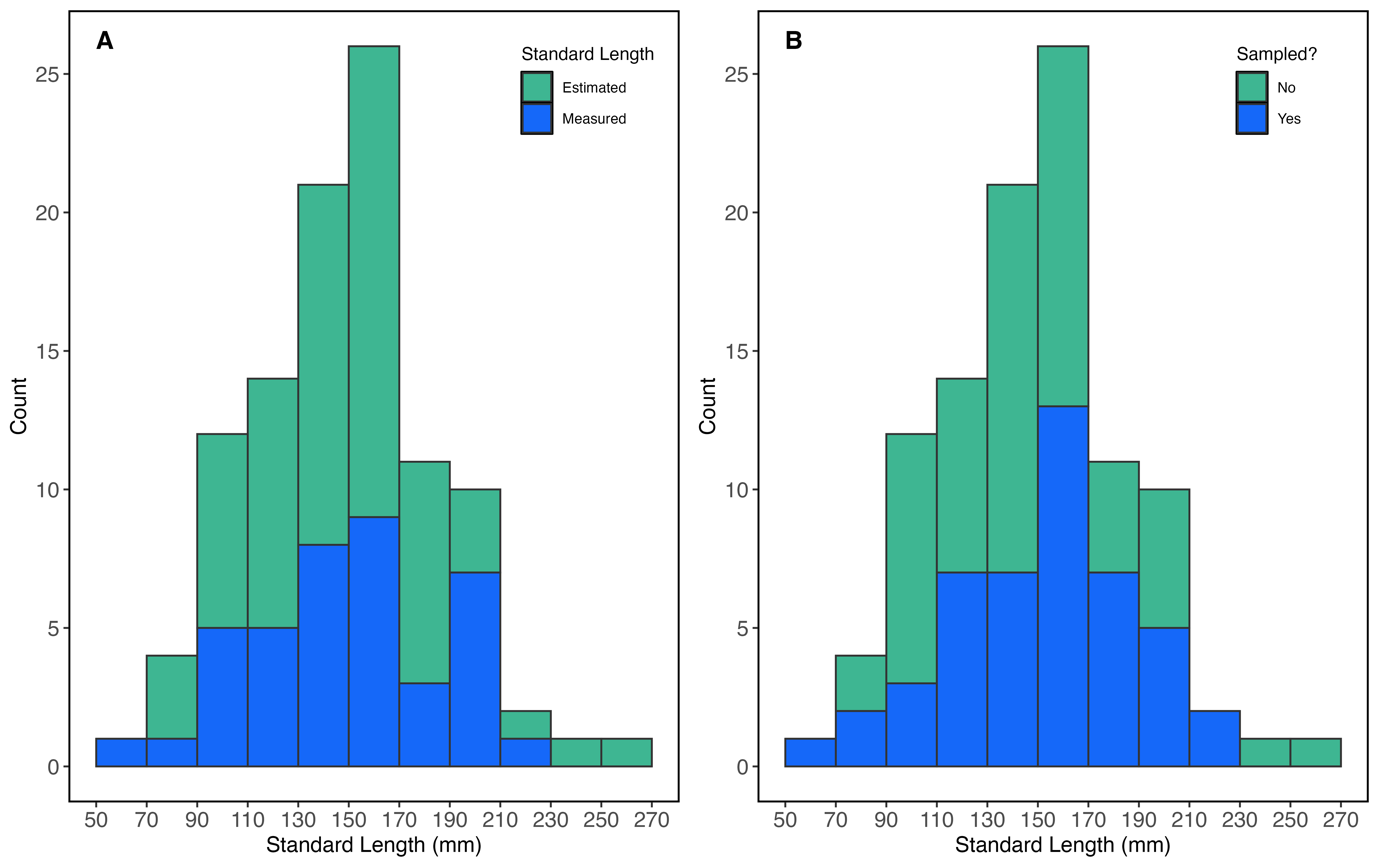


Figure S5: The relationship between 𝛿^18^O-derived seawater temperature estimates and Standard Length (SL) in (A) *Hoplopteryx lewesiensis* and (B) *Hoplopteryx* spp. Here, specimens with SLs smaller than 50% of the maximum SL in our dataset within each 1**°C** temperature band have been removed to account for potential juveniles. Estimated points show the SL generated from a suitable linear regression. Measured points either show the true SL measurement or a mean of two repeats. The error bars on estimated SL points show the standard error of the estimate. No error bars are shown on measured SL points as the smallest and largest values of repeat measurements were smaller than the size of the symbol. Sample sizes are shown on each plot. A regression line (y~x) is added to each plot and the grey-shaded areas represent a 95% confidence interval.


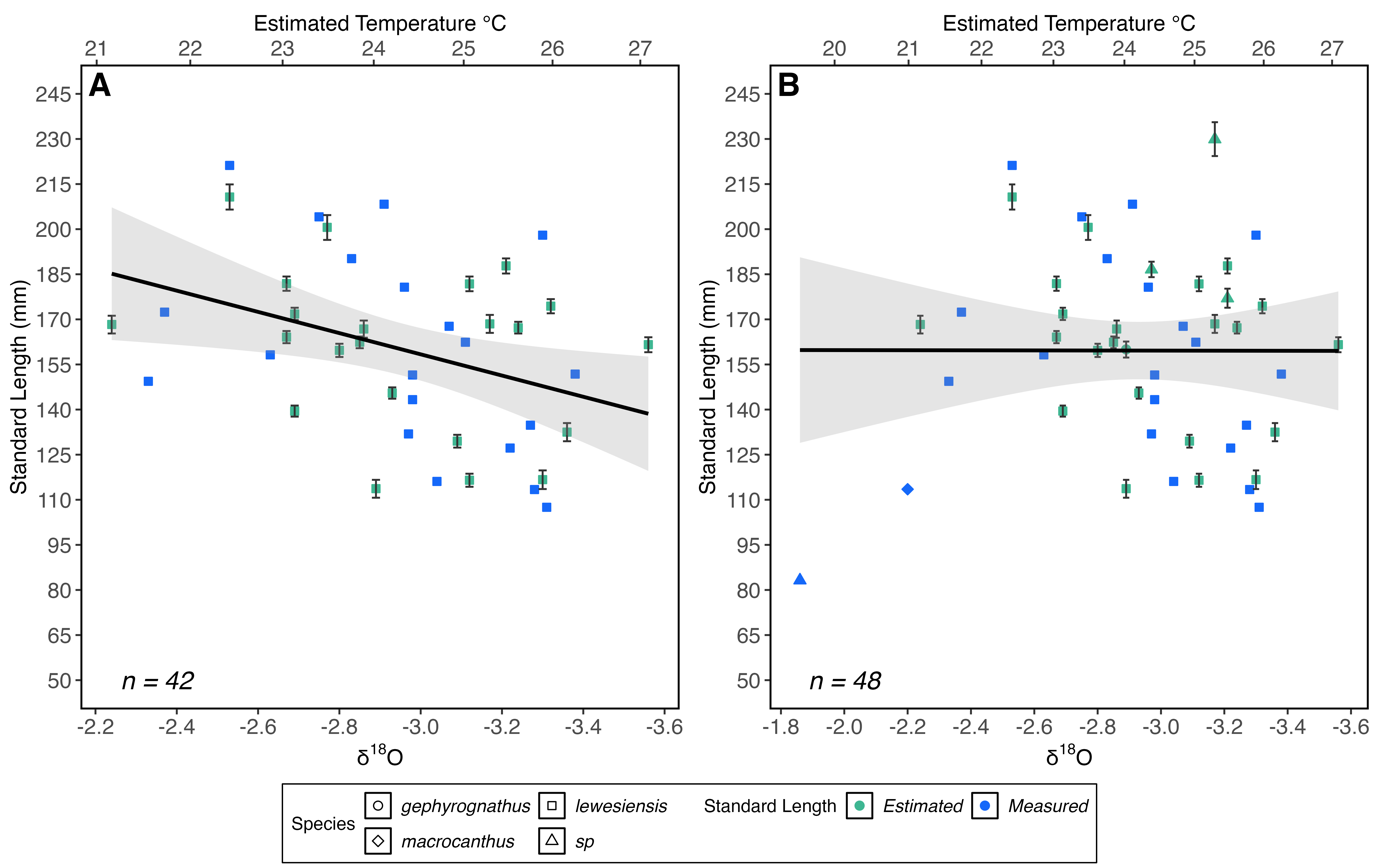


Figure S6: The relationship between 𝛿^13^C and Standard Length (SL) in (A) *Hoplopteryx lewesiensis* and (B) *Hoplopteryx* spp. Here, specimens with SLs smaller than 50% of the maximum SL in our dataset within each 1**°C** temperature band have been removed to account for potential juveniles. Measured points either show the true SL measurement or a mean of two repeats. The error bars on estimated SL points show the standard error of the estimate. No error bars are shown on measured SL points as the smallest and largest values of repeat measurements were smaller than the size of the symbol. Sample sizes are shown on each plot. A regression line (y~x) is added to each plot and the grey-shaded areas represent a 95% confidence interval.

*
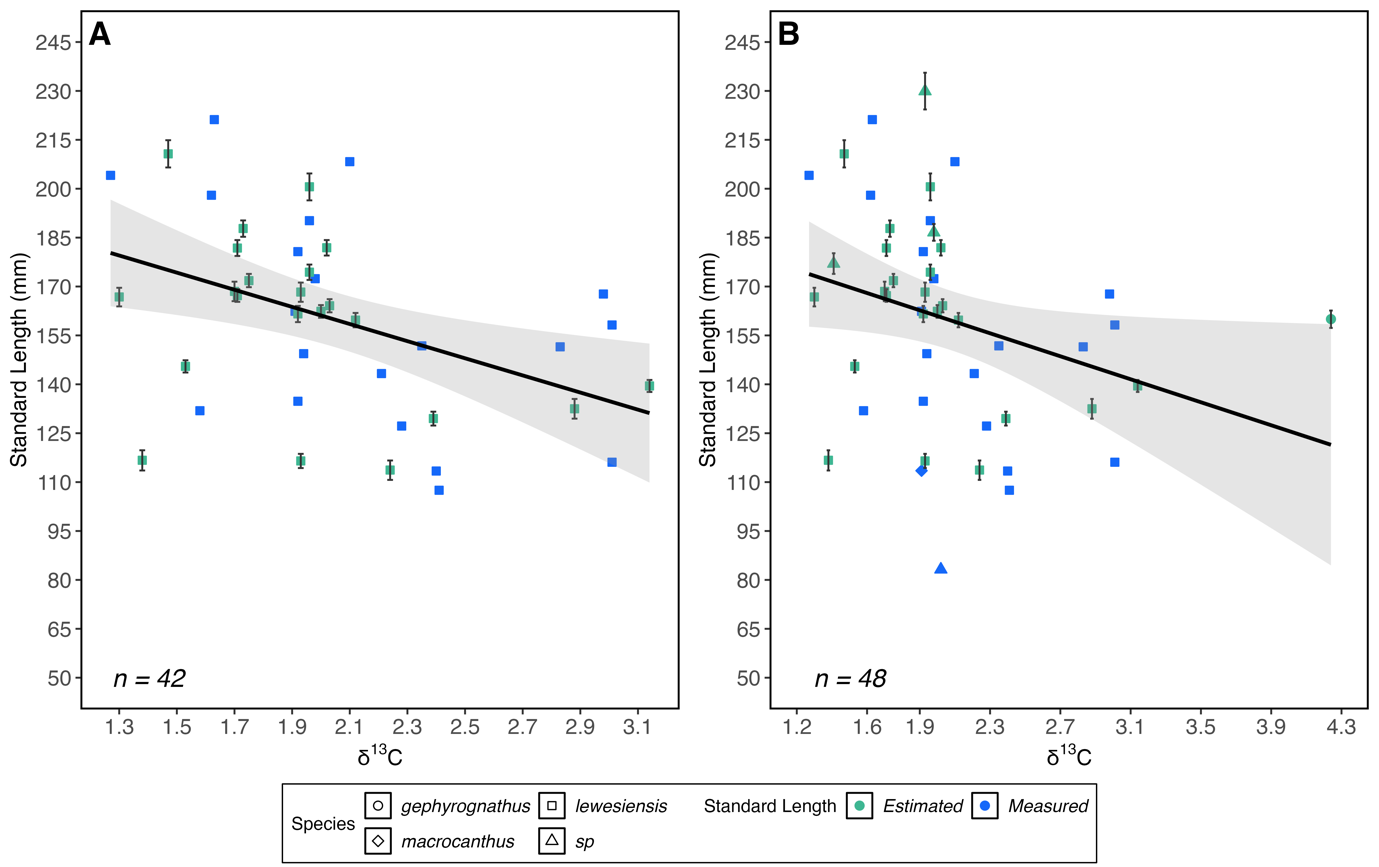
*

# **SUPPLEMENTARY TABLES**

Table S1: Initial and repeat measurements of 23 Hoplopteryx spp. specimens at the Natural History Museum, UK. SL = Standard Length, TL = Trunk Length, HL = Head Length, HH = Head Height, JL = Jaw Length, OD = Orbit Diameter, VW = Vertebrae Width. For each specimen, the top row shows the initial measurement and the bottom shows the repeat.

| NHMUK Registration No. | Species | SL (mm) | TL (mm) | HL (mm) | HH (mm) | JL (mm) | OD (mm) | VW (mm) |
| --- | --- | --- | --- | --- | --- | --- | --- | --- |
| PV OR 79 | *lewesiensis* | 171.36 | 65.38 | 58.73 | 83.10 | 48.37 | 21.30 | 5.70 |
| PV OR 79 | *lewesiensis* | 173.39 | 61.54 | 53.84 | 77.56 | 48.77 | 22.27 | 5.50 |
| PV P 389 | *lewesiensis* | - | 51.77 | 47.98 | 60.83 | 38.30 | 15.70 | - |
| PV P 389 | *lewesiensis* | - | 55.06 | 47.95 | 59.39 | 38.93 | 16.07 | - |
| PV P 1948 | *lewesiensis* | 141.54 | 51.75 | 50.70 | 58.80 | 36.66 | 18.03 | 5.55 |
| PV P 1948 | *lewesiensis* | 141.21 | 56.79 | 48.78 | 57.76 | 37.01 | 17.47 | 5.16 |
| PV OR 4008 | *lewesiensis* | 107.45 | - | 33.51 | 46.02 | 22.21 | 12.17 | 3.52 |
| PV OR 4008 | *lewesiensis* | 107.58 | - | 31.77 | 45.64 | 21.05 | 12.34 | 3.37 |
| PV OR 4014 | *lewesiensis* | 148.79 | - | 41.67 | - | 34.63 | - | 4.16 |
| PV OR 4014 | *lewesiensis* | 150.07 | - | 42.23 | - | 33.72 | - | 4.34 |
| PV OR 4015 | *lewesiensis* | - | - | 59.30 | 66.06 | 38.93 | - | 4.70 |
| PV OR 4015 | *lewesiensis* | - | - | 61.01 | 67.58 | 37.86 | - | 5.03 |
| PV OR 4016 | *lewesiensis* | 149.14 | - | 48.16 | 70.48 | 37.57 | 18.92 | 5.34 |
| PV OR 4016 | *lewesiensis* | 153.95 | - | 48.98 | 65.35 | 37.27 | 19.33 | 5.63 |
| PV OR 4026 | *lewesiensis* | 114.78 | - | 42.70 | 52.01 | 29.00 | 12.01 | 2.31 |
| PV OR 4026 | *lewesiensis* | 111.94 | - | 37.14 | 52.70 | 26.4 | 11.38 | 2.47 |
| PV P 5687 | *lewesiensis* | 199.95 | 79.60 | 68.60 | 89.26 | 52.92 | 20.08 | 7.21 |
| PV P 5687 | *lewesiensis* | 196.09 | 80.55 | 65.57 | 90.27 | 54.62 | 21.37 | 7.04 |
| PV P 5692 | *lewesiensis* | *-* | - | 57.52 | 76.38 | - | 17.51 | 7.55 |
| PV P 5692 | *lewesiensis* | - | - | 53.33 | 72.32 | - | 17.99 | 7.66 |
| PV OR 25781 | *lewesiensis* | 157.68 | - | 51.46 | 70.44 | 43.70 | 19.29 | 4.68 |
| PV OR 25781 | *lewesiensis* | 158.61 | - | 55.22 | 70.54 | 43.72 | 19.00 | 4.34 |
| PV OR 25827 | *lewesiensis* | 81.38 | - | 33.21 | 35.81 | - | 11.00 | - |
| PV OR 25827 | *lewesiensis* | 83.08 | - | 33.44 | 37.27 | - | 10.62 | - |
| PV OR 35712 | *lewesiensis* | 208.49 | - | 70.00 | 71.79 | 56.32 | 21.22 | - |
| PV OR 35712 | *lewesiensis* | 208.16 | - | 68.46 | 68.47 | 55.15 | 21.65 | - |
| PV OR 36917a | *lewesiensis* | 61.38 | - | 23.20 | 26.06 | - | 8.39 | 1.77 |
| PV OR 36917a | *lewesiensis* | 61.91 | - | 23.65 | 26.56 | - | 8.38 | 2.08 |
| PV OR 44836 | *lewesiensis* | 107.60 | - | 34.83 | 55.27 | 35.66 | - | - |
| PV OR 44836 | *lewesiensis* | 108.73 | - | 33.47 | 58.39 | 33.59 | - | - |
| PV OR 49036 | *lewesiensis* | 144.40 | 59.61 | 55.77 | 71.04 | 36.46 | 18.89 | 4.68 |
| PV OR 49036 | *lewesiensis* | 148.94 | 62.59 | 53.02 | 67.56 | 36.49 | 18.99 | 4.96 |
| PV OR 49043 | *lewesiensis* | 220.55 | 105.28 | 65.88 | - | 48.12 | - | - |
| PV OR 49043 | *lewesiensis* | 221.81 | 109.51 | 64.93 | - | 43.92 | - | - |
| PV OR 49865 | *lewesiensis* | - | 61.37 | - | - | - | - | 4.05 |
| PV OR 49865 | *lewesiensis* | - | 57.04 | - | - | - | - | 4.26 |
| PV OR 49888 | *lewesiensis* | *-* | - | 46.49 | 52.08 | 28.59 | - | 3.49 |
| PV OR 49888 | *lewesiensis* | - | - | 50.32 | 50.04 | 28.53 | - | 3.31 |
| PV P 387 | *simus* | *-* | - | 39.35 | 53.95 | - | 14.27 | - |
| PV P 387 | *simus* | - | - | 39.56 | 52.40 | - | 13.94 | - |
| PV P 10222 | *simus* | 85.86 | 26.65 | 23.62 | - | 14.16 | - | - |
| PV P 10222 | *simus* | 87.53 | 23.92 | 26.07 | - | 14.37 | - | - |
| PV P 16971 | sp | *-* | - | 38.77 | 44.41 | - | - | - |
| PV P 16971 | sp | - | - | 38.03 | 44.33 | - | - | - |
| PV OR 49880 | sp | - | - | - | - | - | - | 2.78 |
| PV OR 49880 | sp | - | - | - | - | - | - | 2.89 |

Table S2: Carbon isotope values of International Atomic Energy Agency (IAEA) reference materials, and laboratory standard calcium carbonate. Delta values are on the Vienna Pee Dee Belemnite scale. Uncertainty is shown at the 1-σ level.

|  | δ^13^C, ‰ | | | | | |
| --- | --- | --- | --- | --- | --- | --- |
| Reference material | Assigned value | Uncertainty | Reference | Measured value | Uncertainty | n |
| NBS18 Calcite | -5.04 | 0.06 | Hut 1987, Dunn & Camin 2024. | -5.02 | 0.06 | 7 |
| IAEA-603 Calcite | +2.46 | 0.01 | IAEA | (scale anchor) | 0.03 | 8 |
| IAEA-610 Carbonate | -9.109 | 0.03 | IAEA | (scale anchor) | 0.05 | 8 |
| Calcium carbonate | - | - | - | +1.93 | 0.03 | 25 |

Table S3: Oxygen isotope values of International Atomic Energy Agency (IAEA) reference materials, and laboratory standard calcium carbonate. Delta values are on the Vienna Pee Dee Belemnite scale. Uncertainty is shown at the 1-σ level.

|  | δ^18^O, ‰ | | | | | |
| --- | --- | --- | --- | --- | --- | --- |
| Reference material | Assigned value | Uncertainty | Reference | Measured value | Uncertainty | n |
| NBS18 Calcite | -23.01 | 0.1 | IAEA | -23.06 | 0.16 | 7 |
| IAEA-603 Calcite | -2.37 | 0.04 | IAEA | (scale anchor) | 0.05 | 8 |
| IAEA-610 Carbonate | -18.834 | 0.045 | IAEA | (scale anchor) | 0.10 | 8 |
| Calcium carbonate | - | - | - | -1.94 | 0.08 | 25 |

Table S4: Data collected from 213 Hoplopteryx spp. specimens. SL = Standard Length, TL = Trunk Length, HL = Head Length, HH = Head Height, JL = Jaw Length, OD = Orbit Diameter, VW = Vertebrae Width. BGS = British Geological Survey (Keyworth, UK), CSM = Sedgwick Museum of Earth Sciences (Cambridge, UK), GMZ = Grant Museum of Zoology (London, UK) NHMUK = Natural History Museum (London, UK). Estimated SL are in bold. Italicised SL are the mean of two measurements.

| Institution | Registration No. | Species | SL  (mm) | TL  (mm) | HL  (mm) | HH  (mm) | JL  (mm) | OD  (mm) | VW  (mm) |
| --- | --- | --- | --- | --- | --- | --- | --- | --- | --- |
| BGS | GSd5504-5505 | *lewesiensis* | - | - | - | - | - | 15.1 | 5.2 |
| BGS | GSM109012 | *lewesiensis* | - | - | - | - | - | 15.4 | 4.6 |
| BGS | GSM109076 | *lewesiensis* | - | - | - | - | - | - | 4.8 |
| BGS | GSM109086 | *lewesiensis* | - | - | - | - | - | - | 5.0 |
| BGS | GSM109006 | *lewesiensis* | **132.1** | - | 43.8 | - | 36.1 | 16.4 | 3.6 |
| BGS | GSM109013 | *lewesiensis* | 141.6 | - | - | - | 35.9 | - | - |
| BGS | GSM109082 | *lewesiensis* | 151.2 | - | 48.9 | 68.1 | 42.4 | 19.1 | 4.6 |
| BGS | GSd5503 | *lewesiensis* | **158.6** | - | 52.9 | - | - | - | - |
| BGS | GSM109081 | *lewesiensis* | **160.1** | - | 53.7 | 70.1 | 41.0 | 20.4 | - |
| BGS | GSM109079 | *lewesiensis* | 160.3 | 53.6 | 54.8 | 72.1 | 42.8 | 18.5 | 5.6 |
| BGS | GSM109080 | *lewesiensis* | **170.2** | - | 55.4 | 77.5 | - | - | 6.0 |
| BGS | GSM109078 | *lewesiensis* | **186.9** | - | 61.6 | 83.6 | 48.7 | 17.7 | 7.0 |
| BGS | GSM109007 | *simus* | **89.0** | - | 30.0 | 39.4 | 25.2 | - | - |
| BGS | GSM3020 | *simus* | **151.0** | - | 55.1 | 58.2 | 34.2 | 12.6 | - |
| BGS | GSM109005 | sp | - | - | - | - | - | - | 3.2 |
| BGS | GSM109094 | sp | - | - | - | - | - | - | 2.9 |
| BGS | GSM109098 | sp | **103.5** | - | 34.0 | - | - | - | - |
| CSM | B8963 | *lewesiensis* | - | 68.5 | - | - | - | - | - |
| CSM | B9074 | *lewesiensis* | - | - | - | - | - | - | 7.1 |
| CSM | B94538 | *lewesiensis* | - | - | - | - | - | - | 3.1 |
| CSM | B94512 | *lewesiensis* | **77.0** | - | 26.5 | 33.3 | 19.0 | - | - |
| CSM | B8968 | *lewesiensis* | 100.5 | 32.6 | 34.7 | 48.8 | 28.6 | 19.3 | 3.4 |
| CSM | B8949 | *lewesiensis* | 120.4 | 39.1 | 45.0 | - | - | 15.9 | 5.0 |
| CSM | B8948 | *lewesiensis* | **127.4** | - | 46.1 | 50.0 | - | - | - |
| CSM | B8952 | *lewesiensis* | **129.0** | - | 46.2 | 51.4 | 29.0 | - | 3.1 |
| CSM | B8951 | *lewesiensis* | 129.5 | - | 47.0 | 53.9 | 39.0 | - | 5.0 |
| CSM | B8959 | *lewesiensis* | 135.4 | 45.6 | 46.9 | 64.2 | 32.3 | 16.5 | 4.3 |
| CSM | B8954 | *lewesiensis* | **146.9** | - | 53.5 | 56.7 | 39.5 | 16.3 | - |
| CSM | B8950 | *lewesiensis* | **148.1** | - | - | 65.1 | - | - | - |
| CSM | B8957 | *lewesiensis* | **153.1** | - | 53.4 | 63.3 | - | 17.5 | 4.3 |
| CSM | B8955 | *lewesiensis* | **154.0** | - | - | 67.9 | - | 16.9 | 5.0 |
| CSM | B8953 | *lewesiensis* | **165.3** | - | 59.5 | 64.9 | 35.8 | 18.7 | 4.8 |
| CSM | B8961 | *lewesiensis* | 178.8 | - | 52.2 | 80.0 | 37.2 | 17.3 | 5.7 |
| CSM | B8956 | *lewesiensis* | 198.6 | - | 63.2 | - | - | - | 6.1 |
| CSM | B8958 | *lewesiensis* | 203.1 | - | 74.0 | 94.0 | 50.2 | - | 6.5 |
| CSM | B9125 | *lewesiensis* | **240.0** | - | 80.8 | - | 48.5 | - | 6.2 |
| CSM | B8964 | *lewesiensis* | **262.6** | 115.4 | 94.5 | 102.3 | - | 19.5 | - |
| CSM | B8962 | *lewesiensis?* | - | - | - | - | - | - | 5.5 |
| CSM | B9075 | *lewesiensis?* | - | - | - | - | - | 20.9 | 5.2 |
| CSM | B9104 | *lewesiensis?* | **120.4** | - | 41.1 | 51.7 | 31.3 | 10.9 | - |
| CSM | B9103 | *lewesiensis?* | **127.3** | - | 42.9 | 55.6 | - | 18.3 | - |
| CSM | B9102 | *lewesiensis?* | **152.0** | - | 53.7 | 61.6 | 44.1 | 20.3 | 4.3 |
| CSM | B94511a-b | sp | **56.3** | 20.2 | 17.8 | - | 13.2 | - | 1.2 |
| CSM | B9137 | sp | 181.9 | - | 65.2 | 84.5 | - | 16.6 | 4.8 |
| GMZ | LDUCZ-V920 | *lewesiensis* | 100.2 | 40.9 | 32.9 | 45.5 | 26.6 | 13.7 | 4.1 |
| GMZ | LDUCZ-V2012 | sp | 58.8 | 21.4 | - | - | - | - | 1.8 |
| GMZ | LDUCZ-V2025 | sp | **125.8** | - | 41.7 | - | - | - | - |
| NHMUK | PV OR 41104 | *gephyrognathus* | **160.0** | - | 57.0 | 64.0 | 34.8 | 16.1 | - |
| NHMUK | PV P 3982 | *gephyrognathus* | 181.3 | - | 52.0 | 74.7 | - | 14.4 | 6.9 |
| NHMUK | PV P 10321(a) | *lewesiensis* | - | - | - | - | - | - | 6.0 |
| NHMUK | PV OR 25839 | *lewesiensis* | - | 54.2 | - | - | - | - | 5.3 |
| NHMUK | PV OR 25905 | *lewesiensis* | - | - | - | - | - | - | 5.8 |
| NHMUK | PV OR 25915 | *lewesiensis* | - | - | - | - | - | - | 5.1 |
| NHMUK | PV OR 4027 | *lewesiensis* | - | - | - | - | - | - | 5.6 |
| NHMUK | PV OR 4031 | *lewesiensis* | - | - | - | - | - | 17.6 | - |
| NHMUK | PV OR 4048 | *lewesiensis* | - | - | - | - | 48.4 | - | - |
| NHMUK | PV OR 41105 | *lewesiensis* | - | - | - | - | - | - | 6.2 |
| NHMUK | PV OR 4242 | *lewesiensis* | - | - | - | - | 47.1 | - | - |
| NHMUK | PV OR 49040 | *lewesiensis* | - | - | - | - | - | - | 5.6 |
| NHMUK | PV OR 49041(2) | *lewesiensis* | - | - | - | - | - | 19.5 | 4.3 |
| NHMUK | PV OR 49059 | *lewesiensis* | - | - | - | - | - | - | 4.3 |
| NHMUK | PV OR 49865 | *lewesiensis* | - | *59.2* | *-* | *-* | *-* | *-* | *4.2* |
| NHMUK | PV OR 49866 | *lewesiensis* | - | - | - | - | 31.4 | - | 3.7 |
| NHMUK | PV OR 49872 | *lewesiensis* | - | - | - | - | - | - | 2.6 |
| NHMUK | PV OR 49889 | *lewesiensis* | - | - | - | - | - | - | 3.7 |
| NHMUK | PV P 10035 | *lewesiensis* | - | - | - | - | - | - | 7.7 |
| NHMUK | PV P 10036 | *lewesiensis* | - | - | - | - | - | 28.5 | - |
| NHMUK | PV P 10037 | *lewesiensis* | - | - | - | - | - | - | 8.0 |
| NHMUK | PV P 10038 | *lewesiensis* | - | 65.6 | - | - | - | - | 5.5 |
| NHMUK | PV P 16975 | *lewesiensis* | - | - | - | - | - | - | 2.8 |
| NHMUK | PV P 17313 | *lewesiensis* | - | - | - | - | - | - | 2.9 |
| NHMUK | PV P 17314 | *lewesiensis* | - | - | - | - | - | - | 2.3 |
| NHMUK | PV P 1948(c) | *lewesiensis* | - | - | - | - | - | - | 6.1 |
| NHMUK | PV P 28561 | *lewesiensis* | - | - | - | - | - | - | 4.7 |
| NHMUK | PV P 34443 | *lewesiensis* | - | - | - | - | - | - | 4.0 |
| NHMUK | PV P 36190 | *lewesiensis* | - | - | - | - | - | - | 4.6 |
| NHMUK | PV P 36191 | *lewesiensis* | - | - | - | - | - | - | 6.5 |
| NHMUK | PV P 36236 | *lewesiensis* | - | - | - | - | - | - | 4.9 |
| NHMUK | PV P 5421 | *lewesiensis* | - | - | - | - | - | - | 4.7 |
| NHMUK | PV P 5691 | *lewesiensis* | - | - | - | - | 40.5 | - | 5.1 |
| NHMUK | PV P 5697 | *lewesiensis* | - | - | - | - | 34.2 | - | 3.8 |
| NHMUK | PV P 5698 | *lewesiensis* | - | - | - | - | - | - | 5.0 |
| NHMUK | PV P 6047 | *lewesiensis* | - | - | - | - | - | - | 7.9 |
| NHMUK | PV P 6533 | *lewesiensis* | - | - | - | - | - | - | 7.5 |
| NHMUK | PV P 73754 | *lewesiensis* | - | - | - | - | - | - | 5.3 |
| NHMUK | PV P 77195 | *lewesiensis* | - | - | - | - | - | - | 7.1 |
| NHMUK | PV OR 36917a | *lewesiensis* | *61.6* | *-* | *23.4* | *26.3* | *-* | *8.4* | *1.9* |
| NHMUK | PV P 5423 | *lewesiensis* | **75.7** | - | 24.7 | 35.2 | 19.4 | 10.0 | 2.7 |
| NHMUK | PV OR 25827 | *lewesiensis* | *82.2* | *-* | *33.3* | *36.5* | *-* | *10.8* | *-* |
| NHMUK | PV P 45629 | *lewesiensis* | **88.6** | - | 28.9 | - | - | - | 3.9 |
| NHMUK | PV OR 4012 | *lewesiensis* | 90.1 | - | 27.4 | 37.6 | 22.1 | - | 3.5 |
| NHMUK | PV OR 25918 | *lewesiensis* | **90.8** | - | - | 38.0 | 22.1 | 10.2 | 2.7 |
| NHMUK | PV P 11121 | *lewesiensis* | **91.5** | - | 34.4 | 34.1 | 26.8 | 16.6 | - |
| NHMUK | PV P 6464 | *lewesiensis* | **92.2** | - | 30.3 | 42.1 | 27.7 | 12.0 | 3.1 |
| NHMUK | PV OR 34062 | *lewesiensis* | **98.6** | 35.9 | - | 41.7 | 27.6 | 15.4 | - |
| NHMUK | PV OR 4008 | *lewesiensis* | *107.5* | *-* | *32.6* | *45.8* | *21.6* | *12.3* | *3.5* |
| NHMUK | PV P 6462 | *lewesiensis* | **108.0** | - | 40.8 | 39.5 | 33.5 | 15.1 | - |
| NHMUK | PV P 7394 | *lewesiensis* | **108.0** | - | 38.4 | 43.9 | 31.9 | 14.5 | - |
| NHMUK | PV OR 44836 | *lewesiensis* | *108.2* | *-* | *34.2* | *56.8* | *34.6* | *-* | *-* |
| NHMUK | PV OR 4026 | *lewesiensis* | *113.4* | *-* | *39.9* | *52.4* | *27.7* | *11.7* | *2.4* |
| NHMUK | PV OR 49038 | *lewesiensis* | **113.7** | - | 37.5 | - | - | 16.2 | 3.8 |
| NHMUK | PV P 1948(d) | *lewesiensis* | 116.1 | - | - | - | - | - | 3.5 |
| NHMUK | PV P 1948(a) (1) | *lewesiensis* | **116.5** | - | 39.4 | 50.9 | 33.0 | - | 4.4 |
| NHMUK | PV OR 25912 | *lewesiensis* | **116.7** | - | 42.7 | 45.0 | - | 18.5 | - |
| NHMUK | PV OR 49035(2) | *lewesiensis* | **118.1** | - | 40.5 | 50.5 | 31.3 | 15.0 | 4.3 |
| NHMUK | PV OR 4011 | *lewesiensis* | 127.2 | - | 41.5 | 61.2 | - | 10.8 | 4.9 |
| NHMUK | PV OR 25913 | *lewesiensis* | **127.9** | - | - | 55.6 | - | - | 4.2 |
| NHMUK | PV OR 49039 | *lewesiensis* | **128.7** | - | 43.4 | 56.3 | 30.9 | 14.8 | 3.2 |
| NHMUK | PV OR 25863 | *lewesiensis* | **129.5** | - | 45.2 | 53.8 | - | 21.3 | - |
| NHMUK | PV OR 49041(1) | *lewesiensis* | 131.9 | - | 45.7 | 61.2 | 37.4 | 16.5 | - |
| NHMUK | PV OR 49888 | *lewesiensis* | **132.5** | *-* | *48.4* | *51.1* | *28.6* | *-* | *3.4* |
| NHMUK | PV OR 49867(1) | *lewesiensis* | 134.8 | - | 39.5 | 64.6 | 27.2 | - | 3.7 |
| NHMUK | PV P 1948(a) (2) | *lewesiensis* | **136.0** | - | - | 59.4 | - | 16.5 | 4.4 |
| NHMUK | PV P 10034 | *lewesiensis* | **138.4** | - | 46.0 | - | - | - | 3.8 |
| NHMUK | PV OR 49867(2) | *lewesiensis* | **138.4** | - | 45.3 | 62.8 | - | - | - |
| NHMUK | PV P 5420 | *lewesiensis* | **139.5** | - | 45.6 | 63.4 | 34.2 | 15.7 | - |
| NHMUK | PV P 389 | *lewesiensis* | **140.5** | *53.4* | *48.0* | *60.1* | *38.6* | *15.9* | *-* |
| NHMUK | PV P 6533(a) | *lewesiensis* | **141.0** | - | 48.2 | 60.3 | - | 16.2 | 4.9 |
| NHMUK | PV P 1948 | *lewesiensis* | *141.4* | *54.3* | *49.7* | *58.3* | *36.8* | *17.8* | *5.4* |
| NHMUK | PV P 4842 | *lewesiensis* | 143.3 | 60.2 | 60.5 | 53.5 | 37.5 | 15.5 | - |
| NHMUK | PV P 73791 | *lewesiensis* | **143.8** | - | 49.0 | 61.7 | 37.9 | 16.8 | - |
| NHMUK | PV OR 4019 | *lewesiensis* | **145.5** | - | 47.4 | 66.4 | 36.6 | 15.9 | - |
| NHMUK | PV OR 49036 | *lewesiensis* | *146.7* | *61.1* | *54.4* | *69.3* | *36.5* | *18.9* | *4.8* |
| NHMUK | PV P 9442 | *lewesiensis* | **148.1** | - | 49.7 | 64.8 | 33.8 | 17.6 | - |
| NHMUK | PV OR 4014 | *lewesiensis* | *149.4* | *-* | *42.0* | *-* | *34.2* | *-* | *4.3* |
| NHMUK | PV OR 4016 | *lewesiensis* | *151.5* | *-* | *48.6* | *67.9* | *37.4* | *19.1* | *5.5* |
| NHMUK | PV OR 49870 | *lewesiensis* | 151.8 | 68.0 | 50.1 | 56.7 | 33.6 | 17.8 | - |
| NHMUK | PV OR 42063 | *lewesiensis* | **151.8** | - | - | 66.8 | 29.6 | - | - |
| NHMUK | PV OR 49035(1) | *lewesiensis* | **156.3** | - | 56.0 | 62.0 | 37.9 | 16.9 | 5.3 |
| NHMUK | PV OR 25781 | *lewesiensis* | *158.2* | *-* | *53.3* | *70.5* | *43.7* | *19.1* | *4.5* |
| NHMUK | PV OR 4106 | *lewesiensis* | **159.7** | - | 55.8 | 65.9 | - | 20.6 | 5.1 |
| NHMUK | PV P 7189 | *lewesiensis* | **161.6** | - | 51.4 | 75.7 | 39.4 | 17.7 | - |
| NHMUK | PV OR 25841 | *lewesiensis* | 162.4 | - | 54.6 | 58.5 | 42.1 | 14.5 | - |
| NHMUK | PV P 5690 | *lewesiensis* | **162.4** | - | 53.3 | 73.2 | 33.9 | 18.7 | 5.5 |
| NHMUK | PV P 11822 | *lewesiensis* | **162.7** | - | 55.8 | 69.0 | - | 18.4 | 5.4 |
| NHMUK | PV OR 47300 | *lewesiensis* | **163.4** | - | 54.5 | - | 41.0 | - | - |
| NHMUK | PV OR 41105(a) | *lewesiensis* | **164.1** | - | 53.7 | 74.2 | 42.9 | 21.7 | - |
| NHMUK | PV P 41861 | *lewesiensis* | 165.1 | 68.3 | 50.8 | 74.5 | 40.7 | 18.2 | 5.9 |
| NHMUK | PV OR 4109 | *lewesiensis* | **166.8** | - | - | 73.9 | 51.7 | 24.4 | - |
| NHMUK | PV P 5692 | *lewesiensis* | **167.2** | *-* | *55.4* | *74.4* | *-* | *17.8* | *7.6* |
| NHMUK | PV P 73798 | *lewesiensis* | 167.7 | - | - | - | 39.8 | 14.8 | 4.4 |
| NHMUK | PV OR 4015 | *lewesiensis* | **168.3** | *-* | *60.2* | *66.8* | *38.4* | *-* | *4.9* |
| NHMUK | PV P 5693 | *lewesiensis* | **168.5** | - | 52.9 | 80.3 | - | 19.6 | 6.9 |
| NHMUK | PV OR 49034 | *lewesiensis* | 169.0 | - | - | 79.9 | 38.7 | 20.3 | 6.0 |
| NHMUK | PV P 1948(b) | *lewesiensis* | **171.8** | - | 57.1 | 76.0 | 44.6 | 22.9 | - |
| NHMUK | PV OR 79 | *lewesiensis* | *172.4* | *63.5* | *56.3* | *80.3* | *48.6* | *21.8* | *5.6* |
| NHMUK | PV P 5421b | *lewesiensis* | **172.6** | - | 59.8 | 72.1 | 50.7 | 19.7 | - |
| NHMUK | PV OR 4021a | *lewesiensis* | **174.4** | - | 60.4 | 72.7 | 44.4 | 17.9 | 7.1 |
| NHMUK | PV P 5694 | *lewesiensis* | 180.7 | - | 59.3 | 73.8 | 42.1 | 20.9 | - |
| NHMUK | PV P 5688 | *lewesiensis* | **181.8** | - | 59.7 | 81.7 | - | 22.3 | 6.7 |
| NHMUK | PV OR 49037 | *lewesiensis* | **181.9** | - | 62.1 | 77.4 | 44.2 | 16.9 | - |
| NHMUK | PV P 5689 | *lewesiensis* | **187.8** | - | 63.6 | 80.7 | 46.4 | 23.3 | - |
| NHMUK | PV P 51289 | *lewesiensis* | 190.2 | 73.1 | 63.3 | 91.3 | 54.3 | 21.1 | 6.3 |
| NHMUK | PV P 5687 | *lewesiensis* | *198.0* | *80.1* | *67.1* | *89.8* | *53.8* | *20.7* | *7.1* |
| NHMUK | PV OR 49862 | *lewesiensis* | **200.6** | - | 72.1 | 78.6 | - | 20.3 | 5.9 |
| NHMUK | PV P 388 | *lewesiensis* | **202.5** | - | 70.9 | 82.9 | 47.0 | - | 5.9 |
| NHMUK | PV OR 4030 | *lewesiensis* | **204.1** | - | 73.5 | 79.7 | 49.7 | 18.7 | - |
| NHMUK | PV P 9909 | *lewesiensis* | 204.1 | - | 64.5 | - | 35.9 | - | 7.7 |
| NHMUK | PV OR 49033 | *lewesiensis* | 205.9 | - | 66.1 | 84.3 | 52.7 | 22.8 | 5.6 |
| NHMUK | PV OR 35712 | *lewesiensis* | *208.3* | *-* | *69.2* | *70.1* | *55.7* | *21.4* | *-* |
| NHMUK | PV OR 49863 | *lewesiensis* | **210.7** | - | 74.9 | 84.1 | 53.2 | 20.2 | 5.9 |
| NHMUK | PV OR 49043 | *lewesiensis* | *221.2* | *107.4* | *65.4* | *-* | *46.0* | *-* | *-* |
| NHMUK | PV OR 39074 | *macrocanthus* | - | - | - | - | - | - | 3.5 |
| NHMUK | PV P 12205 | *macrocanthus* | - | - | - | - | - | - | 4.6 |
| NHMUK | PV P 17315 | *macrocanthus* | - | - | - | - | - | - | 2.8 |
| NHMUK | PV P 30186 | *macrocanthus* | - | - | - | - | - | - | 3.7 |
| NHMUK | PV P 48439 | *macrocanthus* | - | - | - | - | - | - | 3.0 |
| NHMUK | PV P 16974 | *macrocanthus* | **97.9** | - | 36.4 | 36.9 | 20.9 | 13.5 | - |
| NHMUK | PV OR 33230 | *macrocanthus* | 113.5 | - | 45.6 | - | 22.3 | - | - |
| NHMUK | PV OR 49074 | *simus* | - | - | - | - | - | - | 4.3 |
| NHMUK | PV OR 49886 | *simus* | - | - | - | - | - | - | 3.7 |
| NHMUK | PV P 5700 | *simus* | - | - | - | - | 29.3 | 14.5 | - |
| NHMUK | PV P 9702a | *simus* | - | - | - | - | - | - | 4.3 |
| NHMUK | PV P 10222 | *simus* | *86.7* | *25.3* | *24.8* | *-* | *14.3* | *-* | *-* |
| NHMUK | PV P 387 | *simus* | **118.9** | *-* | *39.5* | *53.2* | *-* | *14.1* | *-* |
| NHMUK | PV OR 49075 | *simus* | **123.4** | - | - | 53.4 | 28.6 | - | - |
| NHMUK | PV OR 4004 | *simus* | **128.3** | - | 45.6 | 51.9 | - | - | 4.0 |
| NHMUK | PV OR 49073 | *simus* | **130.0** | - | 41.7 | 60.6 | 31.2 | 14.3 | - |
| NHMUK | PV OR 25901 | sp | - | - | - | - | - | - | 2.8 |
| NHMUK | PV OR 4043 | sp | - | - | - | - | - | - | 6.0 |
| NHMUK | PV OR 4047 | sp | - | - | - | - | - | - | 7.6 |
| NHMUK | PV OR 4107 | sp | - | - | - | - | - | - | 4.5 |
| NHMUK | PV OR 4117 | sp | - | - | - | - | - | - | 4.7 |
| NHMUK | PV OR 4121 | sp | - | - | - | - | - | - | 6.6 |
| NHMUK | PV OR 4142 | sp | - | - | - | - | - | - | 5.4 |
| NHMUK | PV OR 49880 | sp | - | *-* | *-* | *-* | *-* | *-* | *2.8* |
| NHMUK | PV P 4293(a) | sp | - | - | - | - | - | - | 2.4 |
| NHMUK | PV OR 44033 | sp | - | - | - | - | 27.5 | - | - |
| NHMUK | PV OR 49061 | sp | - | - | - | - | - | - | 3.8 |
| NHMUK | PV OR 49084 | sp | - | - | - | - | 47.7 | - | - |
| NHMUK | PV P 73792 | sp | - | - | - | - | 39.5 | - | - |
| NHMUK | PV P 76904 | sp | - | - | - | - | - | - | 4.0 |
| NHMUK | PV P 76905 | sp | - | - | - | - | - | - | 4.1 |
| NHMUK | PV P 76906 | sp | - | - | - | - | - | - | 2.7 |
| NHMUK | PV P 76910 | sp | - | - | - | - | - | - | 5.3 |
| NHMUK | PV P 76911 | sp | - | - | - | - | - | - | 4.6 |
| NHMUK | PV P 76912 | sp | - | - | - | - | - | - | 5.1 |
| NHMUK | PV P 76914 | sp | - | - | - | - | - | - | 4.7 |
| NHMUK | PV P 76915 | sp | - | - | - | - | - | - | 4.6 |
| NHMUK | PV P 76913 | sp | **61.8** | - | 21.6 | 26.5 | - | 12.1 | 2.3 |
| NHMUK | PV P 65549 | sp | **79.3** | - | 26.7 | 35.2 | 25.4 | 9.9 | - |
| NHMUK | PV OR 41993 | sp | 83.2 | - | - | - | - | 6.3 | - |
| NHMUK | PV OR 4006 | sp | **93.1** | - | - | 39.1 | - | 15.3 | - |
| NHMUK | PV P 73817 | sp | **102.7** | - | - | 43.6 | - | - | 4.0 |
| NHMUK | PV P 16971 | sp | **108.5** | *-* | *38.4* | *44.4* | *-* | *-* | *-* |
| NHMUK | PV P 73816 | sp | **115.6** | - | 38.1 | - | - | 14.2 | - |
| NHMUK | PV OR 23017 | sp | **128.9** | - | 42.7 | - | 38.5 | - | - |
| NHMUK | PV P 73814 | sp | **130.2** | 48.1 | 41.5 | 61.2 | 34.3 | 15.9 | 4.1 |
| NHMUK | PV P 29992 | sp | **147.2** | - | 47.8 | 67.5 | 30.8 | 17.5 | - |
| NHMUK | PV P 6533(b) | sp | **176.2** | - | 58.9 | - | - | - | - |
| NHMUK | PV OR 28392 | sp | **177.0** | - | 55.7 | 84.0 | 45.3 | 21.7 | - |
| NHMUK | PV P 4297 | sp | **186.6** | - | 64.0 | 78.9 | 42.8 | 20.5 | - |
| NHMUK | PV OR 4239 | sp | **229.9** | - | 83.5 | 88.4 | - | 23.8 | - |
